# *De novo* assembly and annotation of the *Amblyomma hebraeum* tick midgut transcriptome response to *Ehrlichia ruminantium* infection

**DOI:** 10.1371/journal.pntd.0011554

**Published:** 2023-08-14

**Authors:** David Omondi, Erich Zweygarth, Edwin Murungi, Frans Jongejan, Ard M. Nijhof

**Affiliations:** 1 Institute of Parasitology and Tropical Veterinary Medicine, Freie Universität Berlin, Berlin, Germany; 2 Veterinary Centre for Resistance Research (TZR), Freie Universität Berlin, Berlin, Germany; 3 Department of Biochemistry and Molecular Biology, Egerton University, Njoro, Kenya; 4 Department of Veterinary Tropical Diseases, Faculty of Veterinary Science, University of Pretoria, Pretoria, South Africa; 5 Department of Medical Biochemistry, School of Health Sciences, Kisii University, Kisii, Kenya; University of Cincinnati, UNITED STATES

## Abstract

The South African bont tick *Amblyomma hebraeum* is a hematophagous vector for the heartwater disease pathogen *Ehrlichia ruminantium* in southern Africa. During feeding, the tick’s enterocytes express proteins that perform vital functions in blood digestion, including proteins that may be involved in *E*. *ruminantium* acquisition, colonization or immunity. To delineate the molecular mechanism of midgut response to *E*. *ruminantium* infection, we performed comparative analyses of midgut transcriptomes of *E*. *ruminantium* infected engorged *A*. *hebraeum* nymphs, and infected adult male and female ticks with their corresponding matched uninfected controls, before and during feeding. A total of 102,036 unigenes were annotated in public databases and their expression levels analyzed for engorged nymphs as well as unfed and partly-fed adult ticks. There were 2,025 differentially expressed genes (DEGs) in midguts, of which 1,225 unigenes were up-regulated and 800 unigenes were down-regulated in the midguts of infected ticks. Annotation of DEGs revealed an increase in metabolic and cellular processes among *E*. *ruminantium* infected ticks. Notably, among the infected ticks, there was up-regulation in the expression of genes involved in tick immunity, histone proteins and oxidative stress responses. We also observed up-regulation of glycoproteins that *E*. *ruminantium* could potentially use as docking sites for host cell entry. Insights uncovered in this study offer a platform for further investigations into the molecular interaction between *E*. *ruminantium* and *A*. *hebraeum*.

## Author summary

The South African bont tick *Amblyomma hebraeum* is an important vector of heartwater, a debilitating disease of livestock with devastating economic and social impact in sub-Saharan Africa. The causative agent of heartwater is the intracellular proteobacterium *Ehrlichia ruminantium*. To date a single decades-old live vaccine constitutes the main strategy of controlling heartwater infection but is ineffective against heterologous strains of the pathogen in the field. In our study we delineate the molecular mechanism of *A*. *hebraeum* tick midgut genes in response to *E*. *ruminantium* infection. We performed comparative analyses of infected midgut transcriptomes of engorged nymphs, unfed and partly-fed adult ticks with their corresponding age-matched uninfected controls. We present differentially expressed genes that may act as barriers or gateways for *E*. *ruminantium* in tick midgut-cell entry and warrant further investigations in order to develop novel intervention strategies such as anti-tick vaccine candidate molecules. Previous transcriptomic studies have only described *E*. *ruminantium* genes induced during infection *in vitro* and at the tick bite-site or salivary glands.

## Introduction

The South African bont tick, *Amblyomma hebraeum* Koch, 1844 is an obligate hematophagous hard tick that is a vector for *Ehrlichia ruminantium* and *Rickettsia africae*. Both are obligate Gram-negative bacteria responsible for heartwater disease in ruminants and African tick-bite fever in humans respectively [1,2]. It was estimated that heartwater disease infection-fatality accounts for losses of up to US$ 75 million per annum in South Africa alone [3]. The distribution of *A*. *hebraeum* is largely restricted to southern Africa where it mainly parasitizes domestic ruminants (cattle, sheep and goats) and a wide variety of wild ungulates [4,5]. However, the juvenile stages of the vector are indiscriminate in their feeding and parasitize birds and reptiles in addition to small and large ruminants, and may also feed on humans [6]. After accessing the host, *A*. *hebraeum* ticks preferably attach in clusters in the groin, dewlap, belly, udder and interdigital spaces of the feet and when uninterrupted, adult ticks require 6–10 days to fully engorge and detach from the host [7].

*Amblyomma hebraeum* larvae or nymphs acquire *E*. *ruminantium* infection while feeding on an acute or sub-clinically infected host [8]. In the tick midgut, the bacterium undergoes replication within the epithelial cells before spreading to the salivary glands (SG), tick hemocytes and Malpighian tubules [7]. Using electron microscopy, *E*. *ruminantium* reticulated and electron dense inclusion forms were shown to be localized within the cytoplasm of the midgut epithelial cells and acini of the SG [9]. The two pathogen forms are also part of the developmental sequence of *Ehrlichia* in the vertebrate host and *in vitro* cultures. The electron-dense form, apparently infective to cells, survives extracellularly and has been demonstrated to resemble chlamydial organisms [10]. The reticulated form is a predominant vegetative stage as morphologically demonstrated by binary fission in mammalian and invertebrate cells. It is not unambiguously clear if tick transmits the bacterium by regurgitation of the gut contents or via saliva-assisted transmission (SAT) [11]. Gut regurgitation of *E*. *ruminantium* was earlier suggested after homogenates of SG tissues from infected unfed and partly-fed adult ticks did not cause heartwater when intravenously injected into susceptible goats [12]. In subsequent studies, saliva collected from infected ticks was sometimes infective, and SG homogenates were consistently infective to susceptible sheep, suggesting the involvement of SG in *E*. *ruminantium* development. Once the vertebrate host becomes infected, *E*. *ruminantium* appears to infect neutrophils, macrophages and vascular endothelial cells [13]. In acute infection, heartwater is characterized by fever, respiratory distress, gastrointestinal and nervous involvement that precipitates to sudden death [14]. Macroscopical examination of carcasses will uncover hydrothorax, hydropericardium, oedema of the lungs, brain, and splenomegaly [15].

The use of chemical acaricides is the mainstay control strategy for hard ticks such as *Amblyomma* and their associated pathogens. However, the effectiveness of chemical acaricides against *A*. *hebraeum* is hobbled by the ticks’ high reproductive potential, extended longevity without a bloodmeal and broad host spectrum that include wildlife and birds [6,16]. Moreover, chemical acaricides are costly, may pollute the environment, and cause selection of acaricide-resistant tick populations [17]. A single decades-old crude vaccine constitutes the primary strategy of controlling heartwater infection in southern Africa [18]. The technique involves infecting animals with virulent blood containing the Ball 3 strain of *E*. *ruminantium* followed by treatment with antibiotics to prevent severe disease. This procedure is not only a high-risk method, but is impractical and expensive in resource-poor settings of sub-Saharan Africa [19]. Moreover, animal protection with the Ball 3 blood vaccine stock is insufficient to confer immunity against other heterologous *E*. *ruminantium* genotypes [20]. Attenuated and inactivated *E*. *ruminantium* isolates have been shown to protect small ruminants but face safety concerns and are yet to be tested in the field against a natural tick challenge [21–23]. Viable, cost-effective, and environmentally sustainable measures for the control of tick and tick-borne pathogens include use of vaccines that impair tick physiology and/or pathogen transmission [24,25]. These approaches target the tick vector or proteins that play a role in pathogen-vector interactions and can be explored to reduce fitness of the vector or impede pathogen transmission.

Advances in high throughput sequencing technologies have accelerated genomic research [26] and enabled the uncovering of many disease-causing pathogens in ticks [27]. High throughput transcriptomic sequencing has facilitated the determination of pathogen’s and vector tissues’ gene expression during pathogen acquisition and transmission [28,29]. In-depth analysis of midgut transcriptomes provides an avenue for the identifying and development of potential targets for novel tick control methods. Although midgut transcriptomes of an increasing number of tick species have been investigated by high-throughput sequencing [30,31], information regarding *A*. *hebraeum* midgut transcriptome during potential *E*. *ruminantium* infection is lacking.

In this study, Illumina paired-end sequencing was performed and *de novo* assembled transcriptomes of nymphs and adult *A*. *hebraeum* ticks infected with *E*. *ruminantium* compared to transcriptomes of matched uninfected controls. Following functional annotation and classification of the assembly, unigenes involved in tick immunity, oxidative stress responses, and those with possible roles in the *E*. *ruminantium* life cycle within the vector were identified. Collectively, these results provide a foundational platform for further investigations into tick feeding and pathogen colonization that are important to novel intervention strategies such as anti-tick vaccines.

## Materials and methods

### Ethics statement

All animal experiments were conducted with the ethical approval of the local authorities (Landesamt für Gesundheit und Soziales, Berlin, registration number G0240/19).

### Pathogen

The Welgevonden strain of *E*. *ruminantium* isolated in mice from infected *A*. *hebraeum* was obtained from the ARC-Onderstepoort Veterinary Research Institute, South Africa [7]. The pathogen was propagated in bovine endothelial cell lines [32] in Dulbecco’s Modified Eagle Medium/Nutrient Mixture F-12 Ham (Sigma-Aldrich, Taufkirchen, Germany D0547-10X1L, LOT#SLBH5487V) prepared according to the manufacturer’s instruction and supplemented with 10% Gibco fetal bovine serum (REF#10270–106, LOT#2440090—Thermo Fisher Scientific, Waltham, US) and 1% penicillin/streptomycin (Thermo Fisher Scientific).

### Ticks

A*mblyomma hebraeum* nymphs previously collected in KwaZulu-Natal province, South Africa were received from the Utrecht Centre for Tick-borne Diseases. Before use, a proportion of the nymphs were screened to confirm the absence of *E*. *ruminantium* infection by amplifying the major antigen protein 1 (*map*-1) gene. Briefly, 20 nymphs were picked randomly, individually left to soak and soften in an Eppendorf tube with 200 μL PBS before being homogenized mechanically using a disposable pestle. DNA was extracted from the resulting homogenate using the NucleoSpin tissue kit as per the manufacturer’s instructions (Macherey-Nagel, Düren, Germany). A 669-bp fragment of the *E*. *ruminantium* (*map-*1) gene was subsequently amplified using primers Er_MAP1_F1_1127 (5’-CAAAATACAACCCAAGCATACCAC-3’) and Er_MAP1_R2_1796 (5’- GCGTCAAGAGTTACTGAAGCGG-3’). The PCR was performed in a 25 μL reaction volume consisting of 0.5 U Fusion S7 polymerase (Mobidiag), 5 μL 5 × HF buffer, 200 μM of each dNTP, and 10 pmol of each primer. Cycling conditions were 98°C for 30s, 40x (98°C for 10s, 63°C for 20s, 72°C for 25s), followed by a final extension step at 72°C for 10 min.

### Animals

The sheep (9–12 months old) involved in the study were purchased locally and confirmed PCR-negative for *E*. *ruminantium* by intravenous withdrawal of blood sample, followed by total DNA extraction using the NucleoSpin DNA blood kit as per the manufacturer’s instructions (Macherey-Nagel, Düren, Germany). The protocol for amplification of the *E*. *ruminantium* (*map*-1) gene was similar as described above. The animals were kept in a controlled animal stable at the Institute of Parasitology and Tropical Veterinary Medicine, Berlin, Germany.

### Experimental design

**Fig 1:**
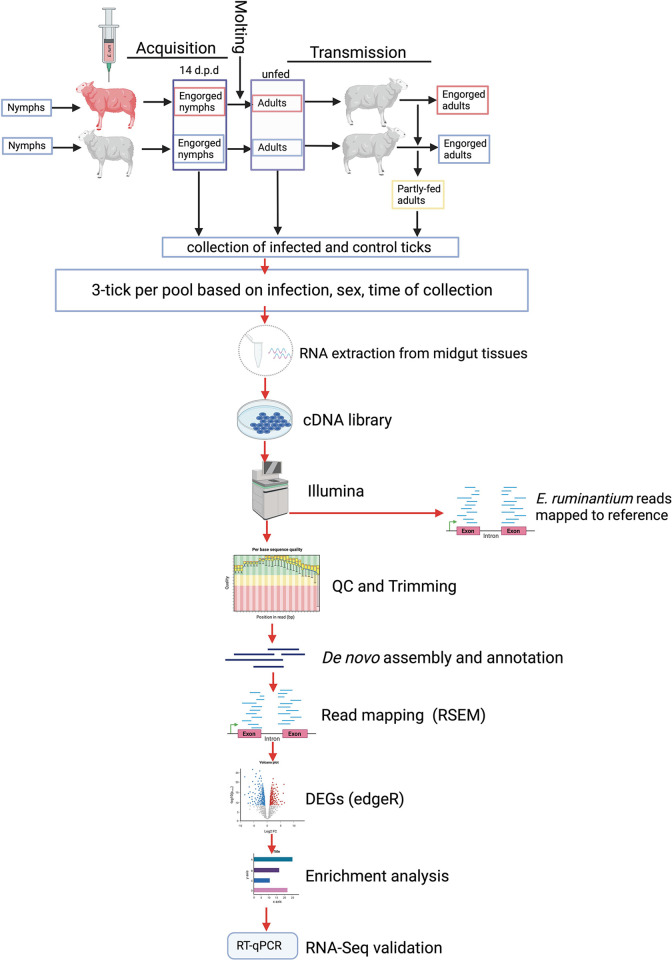
Schematic representation of the experimental design showing *E*. *ruminantium* acquisition and transmission by *A*. *hebraeum* ticks as well as the downstream analysis of the resultant tick midgut transcriptomes by RNA-seq analysis. The figure was created and exported under a paid subscription with BioRender.com.

We tested the acquisition of the Welgevonden strain of *E*. *ruminantium* by *A*. *hebraeum* nymphs using RNA-seq (**Fig 1**). A nine-month-old sheep was inoculated intravenously with 1.5 mL *E*. *ruminantium* infected bovine endothelial cell culture supernatant as previously described [32]. Rectal temperatures of the sheep were monitored daily, along with associated heartwater clinical symptoms. Nymphs were allowed to feed on the sheep in ear bags from day two and four post-inoculation onwards, before the rectal temperature had increased above 39.5° C. All nymphs were allowed to engorge to repletion. Age-matched nymphs were simultaneously allowed to feed on a control sheep that was not inoculated with *E*. *ruminantium*. A proportion of fully engorged nymphs (day 14 post detachment) were processed for RNA-seq while the remaining nymphs were allowed to molt. Four months after acquisition, adult ticks from both groups were allowed to feed on naïve sheep. The midguts of adult ticks were collected at different time points: from unfed females and males at day 0, partly-fed males at 48-h (day 2) post attachment and from partly-fed females at 72-h (day 3) post attachment as shown in **Fig 1**.

### Tick dissection and midgut and SG collection

*Amblyomma hebraeum* tick midguts were dissected from engorged nymphs (14 days post-detachment), unfed and partly-fed adult ticks within the first hour of tick collection. The midgut tissues were collected using ultra-fine forceps and rinsed in ice-cold PBS, pH 7.4 before being transferred to Eppendorf tubes containing 300 μl Tri reagent (Thermo Fisher Scientific, Waltham, US) on ice. The tissues were then pooled from three individual ticks and in three biological replicates per stage, sex and time point of tick collection, and stored in -80°C until RNA extraction. **Fig 1**.

### RNA extraction, quantification and integrity

Midguts stored in Tri reagent were thawed and homogenized by repeatedly passing through 25G needles. Fresh Tri reagent was added to the homogenate to a final volume of 500μL. RNA was then isolated from the homogenate by adding 100 μl chloroform, followed by vigorous vortexing, incubation at room temperature (RT) for 15 min and centrifugation at 12,000 g for 15 min at 4°C. The RNA-containing aqueous phase was further purified on Direct-zol RNA miniprep Plus columns (Zymo Research) following the manufacturer’s protocol that included a DNase digestion step. The RNA was further purified using the NucleoSpin RNA Clean-up XS (Macherey-Nagel) according to the manufacturer’s instructions. The RNA concentration was quantified using a Nanodrop spectrophotometer and further assessments to determine integrity and purity were done using agarose gel electrophoresis.

### cDNA library preparation and sequencing

The library preparation process was done by isolation of mRNA from the total RNA sample using magnetic beads of oligos d(T)25, a method referred to as polyA-tailed mRNA enrichment. The mRNA was subsequently randomly fragmented and reverse transcribed into cDNA using random hexamers and reverse transcriptase. Following the completion of the first strand synthesis, the second strand was produced by incorporating an Illumina buffer, dNTPs, RNase H, and *Escherichia coli* polymerase I through a process known as Nick translation. Next, the resultant cDNA underwent a series of steps; purification, end-repair, a-tailing, and adapter ligation. The appropriately sized fragments were then enriched via PCR, during which indexed P5 and P7 primers were introduced. The final products underwent a final purification step. Each sample was then subjected to meta-transcriptomic analysis through sequencing, which generated an estimated 12 Gb of raw data, equivalent to ~40 million paired-end reads. We used a paired-end 150bp (PE150) strategy to sequence the *A*. *hebraeum* midgut meta-transcriptome in a Novaseq6000 system. During the library preparation phase, we targeted a cDNA insert size ranging between 250 and 300 base pairs. Ribosomal depletion was performed using the Illumina Ribo-Zero Plus rRNA Depletion Kit to minimize the high abundance of rRNA molecules, thereby enhancing the detection of functionally relevant coding and non-coding transcripts from both tick and bacterial sources. Following rRNA depletion, we synthesized the first and second strands of cDNA from the purified mRNA using the NEBNext Ultra Directional RNA Library Prep Kit, strictly adhering to the manufacturer’s guidelines. The sequencing process was done by Novogene, China.

### Data filtering

The Illumina sequencing raw data was transformed into FASTQ format using CASAVA v1.8. The quality of the sequence reads was evaluated using FastQC v0.11.9 (http://www.bioinformatics.babraham.ac.uk/projects/fastqc/) and adapter sequences removed using Trimmomatic v3.8 [33]. Further, reads containing > 10% unknown nucleotides (N) and those with a quality score (Qscore) of < 5 for over 50% of the bases were removed. The quality of the clean reads was reassessed using FastQC and the forward or reverse components of the clean paired reads from different treatments were then concatenated.

### *De novo* transcriptome assembly and quality assessment

Concatenated clean reads were assembled into transcripts using the short read *de novo* assembler Trinity v2.8.4 [34] in a *de novo* RNA-Seq Assembly Pipeline (DRAP) [35]. Trinity compiles the raw reads data into several de Bruijin graphs and ultimately reports transcripts (contigs) in their final form. DRAP compacts and error corrects the Trinity assembly by removal of sequence redundancies and cluster the contigs based on longest ORF and CD-HIT[36]. The completeness and quality of the assembled transcriptomes was evaluated using BUSCO version 5.1.2 with the arthropoda_odb10 lineage dataset (created on 2020-09-10, with 90 genomes and 1013 BUSCOs) [37] and QUAST [38] respectively. BUSCO performs a quantitative evaluation and annotation of completeness of an assembly while assembly assessment by QUAST involves computation of various quality metrics including number of contigs and median contig length.

### Functional annotation of *A*. *hebraeum* unigenes

Assembled unigenes were annotated by querying them against NCBI-NR, Pfam, Swiss-Prot, COG and GO databases. The database searches were performed using BlastX v2.9.0 [39] with an E-value cut-off of 1e-05. Querying of Pfam database [40] for protein domain annotation was undertaken using HMMER v3.1 [41] with an E-value cut-off of 1e-02. Gene ontology (GO) terms were assigned to the transcripts by scanning the GO database using Blast2GO [42] with an E-value cut-off of 1e-06. To visualize specific pathways that the unigenes are involved in, the unigenes were mapped onto the Kyoto Encyclopedia of Genes and Genomes (KEGG) with an E-value cut-off of 1e-05. To delineate the transcripts orthologous relationships, COG database [43] was queried using BlastX at an E-value of 1e-05.

To disentangle the *E*. *ruminantium* reads from the rest of the transcriptome, we mapped all the reads to *E*. *ruminantium* reference genome [44]. Before paired-end clean reads were mapped, the reference genome and gene model annotation files were downloaded and indexed using Hisat2 v2.0.5. The mapped reads of each sample were assembled by StringTie (v1.3.3b) [45] in a reference-based approach and assembly statistics computed.

### Gene expression analysis of *A*. *hebraeum* transcriptome

To determine the level of gene expression, sequence reads were mapped onto the filtered transcriptome using Bowtie 2 [46] and the mapping results analyzed using RSEM [47]. These computations were done in General-purpose High-Performance Computer at the Freie Universität Berlin [48]. RSEM quantifies the read count for each gene in each sample and converts this metric into TPM (Transcript per Million base pairs sequenced) values. While counting fragments, TPM considers the effects of sequencing depth and gene length. TPM distribution plots were used to compare gene expression levels in different conditions. Correlation between samples was determined by calculating the correlation coefficient (R^2^), the square of the Pearson coefficient, which enables ascertaining similarity at gene expression level. A higher correlation means higher similarity and minimal number of DEGs.

### Differential expression and enrichment analysis

Read counts obtained from gene expression analysis were used to identify DEGs using edgeR [49]. First, the counts were normalized and p-value estimated using a negative binomial distribution model. FDR (false discovery rate) was then estimated based on multiple hypothesis testing. Genes were presumed to be differentially expressed if |log2(Foldchange) | ≥ 1.5 and qval < 0.05. Differentially expressed genes (DEGs) were mapped onto the KO database to ascertain KEGG enriched pathways for the DEGs determined by calculating the Rich factor and q-value. Rich factor is the ratio of DEGs to the total annotated genes in a pathway while q-value is the normalized p-value.

### Experimental validation of transcriptome data by RT-qPCR

The transcriptome data was validated by selection of at least 8 DEGs of top up-regulated and down-regulated from each comparison in the nymph, unfed and partly-fed adult tick transcriptome libraries by RT-qPCR. The cDNA was synthesized from aliquots of the RNA samples that were previously shipped for RNA-seq along with their corresponding biological duplicates using PhotoScript II First Strand cDNA Synthesis Kit (New England BioLabs Inc, Ipswich, US) according to manufacturer´s instruction. The qPCR reactions were performed using Luna Universal qPCR Master Mix (New England BioLabs Inc) according to manufacturer’s protocol. Primers for qPCR assay were designed by first locating the open-reading frame (ORF) of the DEG using the NCBI open reading frame finder followed by Primer-BLAST tool of NCBI [50]. The list of primers generated for the validation of RNA-seq data is presented in **S1 Tables**. E6rcfvTGB/Hnjach reaction was performed in three technical replicates and the *A*. *hebraeum* translation elongation factor 1 alfa (GenBank accession number AF240836) was used as a reference gene for normalization of the qPCR assays. The expression levels of the selected DEGs were calculated using the 2^–∆∆Ct^ method, followed by the comparison of Log_2_fold change between RNA-seq and qPCR [51].

## Results

### *E*. *ruminantium* infection in experimental animals

**Fig 2:**
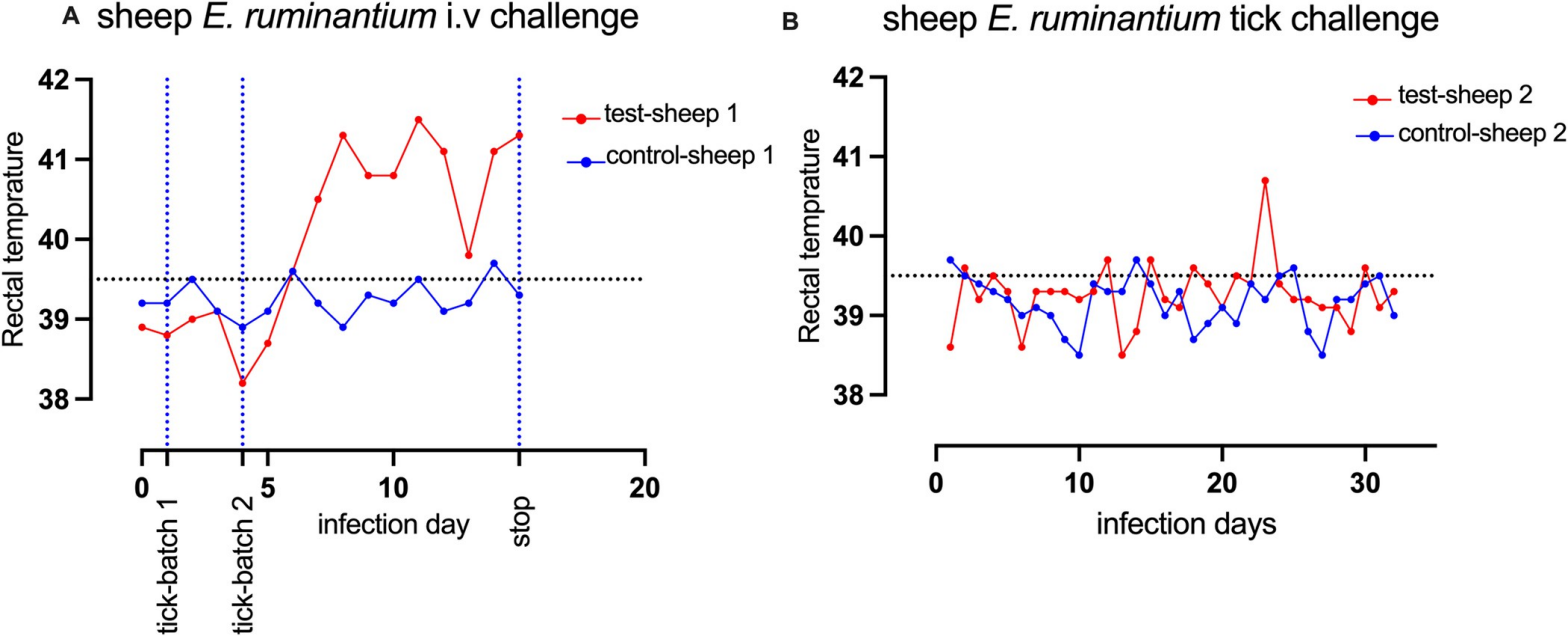
Monitoring of rectal temperatures in experimental and control sheep during *E*. *ruminantium* acquisition and transmission by *A*. *hebraeum* ticks. Panel A shows the monitored rectal temperatures of sheep during the acquisition of *E*. *ruminantium* by *A*. *hebraeum* nymphs. The test sheep (depicted in red) was administered an intravenous inoculum of *E*. *ruminantium* at day 0, while the control sheep (depicted in blue) did not receive an inoculum. Two batches of *A*. *hebraeum* nymphs were allowed to feed on both sheep on days 2 and 4 post-inoculation. The experiment concluded at day 15, following the nymphs’ completion of feeding. Panel B displays the rectal temperatures of a new set of naïve sheep during *E*. *ruminantium* transmission by the emerging *A*. *hebraeum* adults. Adults potentially infected with *E*. *ruminantium* were allowed to feed on test-sheep 2 to mimic a natural transmission, while those from the control-sheep 1 fed on control-sheep 2 (shown in blue).

The sheep experimentally infected intravascularly by *E*. *ruminantium* developed signs of heartwater, including lethargy and fever from day 6 to 15 when the experiment was terminated and the sheep euthanized **(Fig 2A)**. Other notable symptoms observed were labored breathing and recumbency. Two batches of 80 nymphs fed on the infected sheep to repletion of which 134 adults molted (60 females and 74 males), representing an 83.8% molting rate. In the control sheep, 86 engorged nymphs resulted in 41 males and 28 females, representing 80.2% molting rate. The emerged adults (n = 40) that had fed as nymphs on the *E*. *ruminantium* infected sheep were potentially infective and were allowed to feed on a naive sheep to mimic the natural transmission of *E*. *ruminantium*, as it would occur in the field. However, a febrile reaction was not observed in the second test sheep, and the detection of *E*. *ruminantium* in both blood and brain samples were negative, suggesting a lack of *E*. *ruminantium* transmission to the naïve sheep by adult tick feeding **(Fig 2B)**. All midgut pools of ticks that had fed on infected animal tested PCR-positive for *E*. *ruminantium* and showed disparities in the number of *E*. *ruminantium* specific sequence reads among groups. Unfed males had the highest number of *E*. *ruminantium* sequence reads in the midgut (663,527 reads) while partly-fed female had the least (2,860 reads) (**Table 1)**.

**Table 1:** Mapping statistics of *E*. *ruminantium* reads in infected midgut groups of *A*. *hebraeum* ticks.

Sample name	Unfed-females	Fed-females	Unfed-males	Fed-males	nymphs
Total reads	94,456,800	101,710,438	87,718,886	82,281,824	92,786,072
Total mapped	32,318 (0.03%)	2,860 (0.00%)	663,527 (0.76%)	7,484 (0.01%)	4,561 (0.00%)
Multiple mapped	57 (0.00%)	10 (0.00%)	2,445 (0.00%)	13 (0.00%)	16 (0.00%)
Uniquely mapped	32,261 (0.03%)	2,850 (0.00%)	661,082 (0.75%)	7,471 (0.01%)	4,545 (0.00%)
Forward reads	16,213 (0.02%)	1,420 (0.00%)	331,366 (0.38%)	3,751 (0.00%)	2,262 (0.00%)
Reverse reads	1,6048 (0.02%)	1,430 (0.00%)	32,9716 (0.38%)	3,720 (0.00%)	2,283 (0.00%)
Reads map to ’+’	16,070 (0.02%)	1,424 (0.00%)	330,136 (0.38%)	3,731 (0.00%)	2,282 (0.00%)
Reads map to ’-’	16,191 (0.02%)	1,426 (0.00%)	330,946 (0.38%)	3,740 (0.00%)	2,263 (0.00%)
Non-splice reads	32,116 (0.03%)	2,834 (0.00%)	656,270 (0.75%)	7,450 (0.01%)	4,540 (0.00%)
Splice reads	145 (0.00%)	16 (0.00%)	4,812 (0.01%)	21 (0.00%)	5 (0.00%)

### *De novo* assembly statistics

An estimated 927 million reads were obtained from midgut tissues. After normalization, 143 million clean reads were used to generate the assembly. Sequencing statistics of the midgut samples are shown in **Table 2**. Following the removal of adapter sequences, low quality, and ambiguous reads, *de novo* assembly of reads resulted in 571,913 transcripts with an N50 of 3,196 bp and 102,036 unigenes with an N50 of 3,815 bp see **Table A in S2 Tables**. The mapping back statistics for the sample reads on the indexed unigenes is also presented in **Table B in S2 Tables**. The 102,036 unigenes had a high level of completeness, with 94.0% (953 of the 1013 BUSCO groups searched) being complete. This consisted of 59.2% being single-copy BUSCOs and 34.8% being duplicates. A minor fraction, 2.4%, of the BUSCOs were fragmented while only 3.6% were missing from the assembly.

**Table 2:** Statistical summary of *Amblyomma hebraeum* midgut sequencing of ten RNA samples in five comparison groups.

Sample	Raw reads	Clean reads	Error rate	Q20	Q30	GC (%)	
Nymph midgut pos	46,679,800	46,286,305	0.03	96.91	92.19	52.53	
Nymph midgut neg	47,319,799	46,748,569	0.03	97.03	92.73	52.77	
Unfed-male midgut pos	44,281,921	43,728,923	0.03	96.68	91.91	50.5	
Unfed male midgut neg	47,539,555	46,993,528	0.03	96.71	92.06	50.49	
Partly-fed male midgut pos	41,478,216	41,045,565	0.03	96.98	92.14	50.73	
Partly-fed male midgut neg	41,833,626	41,503,712	0.03	96.98	92.14	50.23	
Unfed female midgut pos	47,585,496	47,102,720	0.03	96.71	91.68	49.34	
Unfed female midgut neg	45,680,511	45,219,064	0.03	96.76	91.72	49.48	
Partly-fed female midgut pos	60,423,710	59,971,579	0.03	96.33	91.38	52.97	
Partly-fed female midgut neg	41,012,690	40,690,708	0.03	96.82	91.93	51.35	

^pos^ midgut was confirmed positive for *E*. *ruminantium* infection. ^neg^ midgut of control group

### Functional annotation and classification of *A*. *hebraeum* transcriptome

**Fig 3:**
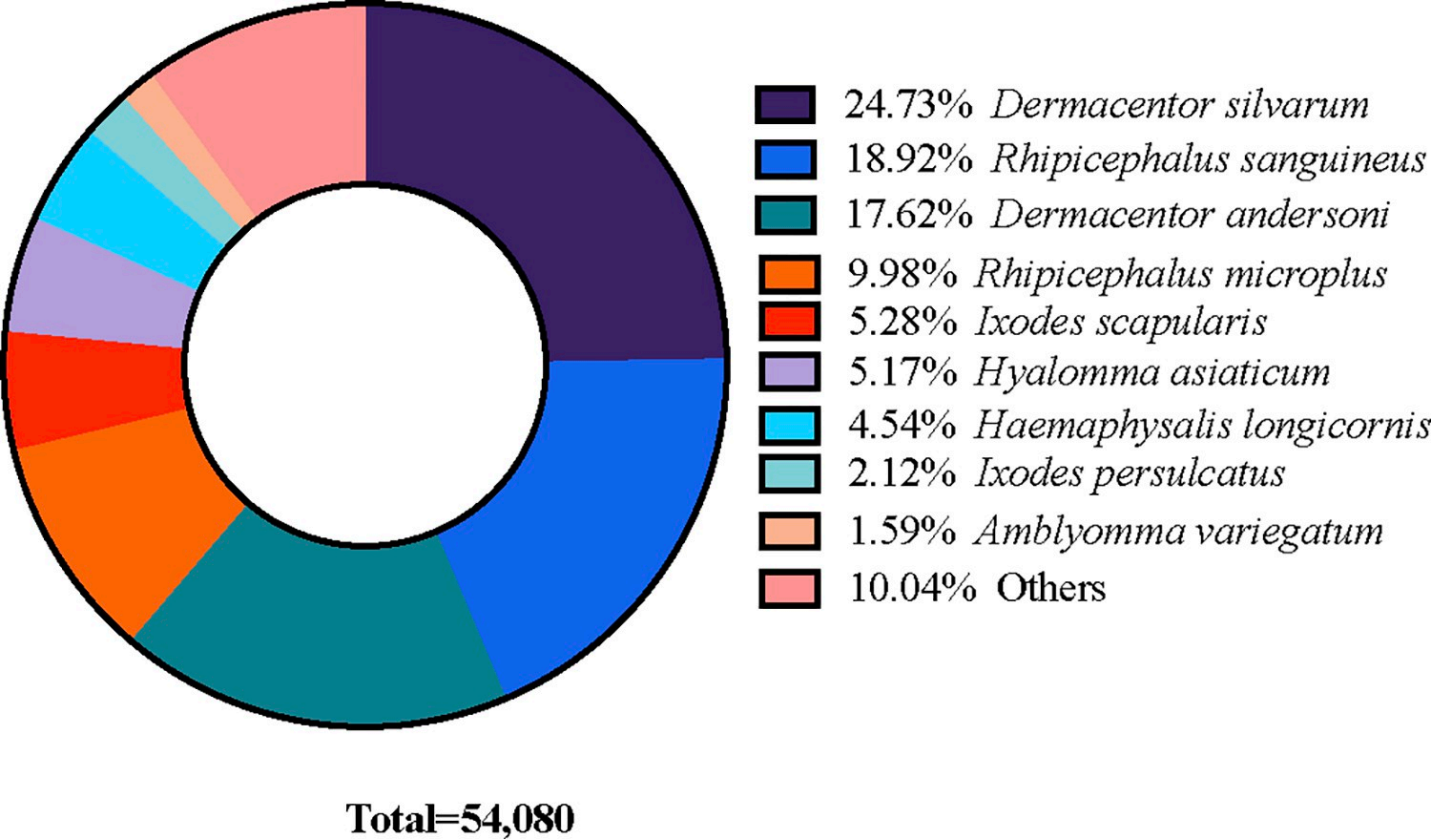
Species classification of BLASTx matches: A pie chart representation of *A*. *hebraeum* transcriptome unigenes.

There were a total 102,036 unigenes obtained from the *A*. *hebraeum* midgut transcriptome assembly. Of these, 54,080 unigenes (53.01%) and 23,090 unigenes (22.63%) were annotated in NCBI-NR and SwissProt databases respectively. The number of unigenes annotated in other major databases are also presented in **Table C in S2 Tables**. According to the top-hit species distribution in the NCBI-NR database, most matches were found with the Taiga tick *Dermacentor silvarum*, accounting for 13,373 unigenes (24.73%) of the total. This was closely followed by 10,234 unigenes (18.92%) that aligned with the brown dog tick *Rhipicephalus sanguineus*, and 9,529 unigenes (17.62%) that corresponded to the Rocky mountain wood tick *Dermacentor andersoni*. The distribution of the top ten species matches in the NCBI-NR database is presented in **Fig 3**.

We annotated a total of 3,317 unigenes under the three broad Gene Ontology (GO) categories of molecular function, biological process, and cellular component. The molecular function was the most abundant category with 1,263 unigenes; 38.34% of the total. The biological process category accounted for the second most abundant set with 1,113 unigenes; 33.79% and the cellular component category contained the least, with 918 unigenes representing 27.87%. Within the biological process category, ’cellular process’ (894 unigenes) and ’organic substance metabolic process’ (793 unigenes) were the most represented GO terms, while ’translation’ (111 unigenes) was the least represented. In the cellular component category, ’cellular anatomical entity’ (900 unigenes) and ’organelle’ (589 unigenes) were the most common terms, and ’plasma membrane’ (91 unigenes) was the least common. Finally, within the molecular function category, ’binding’ (749 unigenes) and ’heterocyclic compound binding’ (737 unigenes) were the most represented terms, while ’ATP binding’ (142 unigenes) was the least represented. The distribution of all the GO terms as well as 6,995 unigenes annotated by COG database is illustrated in **S1 Fig.**

### Differential gene expression analysis

**Fig 4:**
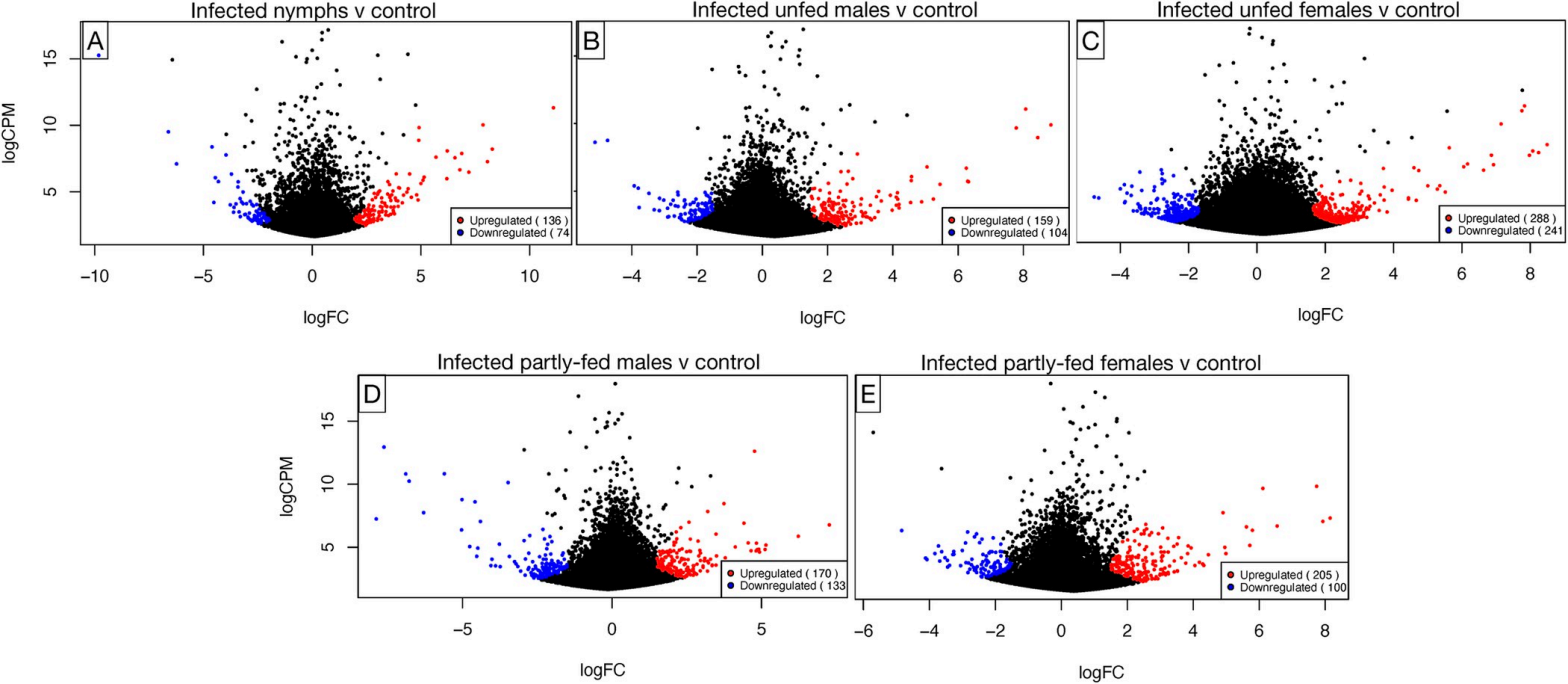
Differential gene expression analysis in *E*. *ruminantium* infected *A*. *hebraeum* midguts visualized as a volcano plot. This plot compares the DEGs across five *E*. *ruminantium* infected *A*. *hebraeum* midgut groups to respective controls. Red dots signify significantly up-regulated genes, blue dots represent significantly down-regulated genes, and blank points denote genes with no significant change in expression. Significance was determined based on a log_2_FC ≥ 1.5 or log_2_FC ≤ -1.5 and a q-value < 0.05. The y-axis plots the logCPM values, providing a measure of each gene’s expression level (Counts Per Million).

We conducted a comparative analysis of differentially expressed genes (DEGs) across various midgut groups of engorged nymphs, both infected and control and across adult males and females tick at different feeding stages (infected unfed at day 0, partly-fed at day 2 for males, and day 3 for females), and their respective controls(see **Table 2**). In the nymph midgut transcriptome, we identified a total of 210 DEGs. Of these, 136 unigenes were up-regulated and 74 unigenes were down-regulated (**Fig 4A**). We observed an up-regulation of genes associated with the immune system and oxidative stress response in *E*. *ruminantium*-infected nymphs compared to the control group. Notably, several unigenes were highly up-regulated in the nymph midgut suggesting their possible roles in tick immunity or pathogen-host interaction. These included antimicrobial peptides, such as acanthoscurrin 1 and 2- like proteins, holotricin 3- like and lectins. Other known significantly up-regulated genes were associated with ecdysis and enzymes critical to metabolic processes. Top DEGs up-regulated in the infected nymph midgut are presented in **Table 3**. The full set of significantly up-regulated and down-regulated DEGs in nymph midgut is detailed in **S1 Data**.

**Table 3:** Selected top DEGs up-regulated in *E*. *ruminantium* infected *A*. *hebraeum* nymph midgut tissues at 14 days post detachment from host.

Unigene ID	TPM_Pos	TPM_Neg	log2FC	logCPM	FDR	Blast_nr Annotation
trinity_SMG_CL5318Contig2_1	13,931.53	5.84	11.28	11.35	1.65E-02	XP_037268604.1 acanthoscurrin-2-like [*Rhipicephalus microplus*]
trinity_SMG_CL77607Contig1_1	1,326.22	2.41	9.12	7.96	6.52E-03	XP_054934363.1 uncharacterized protein LOC126543383 [*Dermacentor andersoni*]
trinity_SMG_CL233Contig5_1	720.28	1.78	8.65	7.09	3.69E-03	XP_037564726.1 acanthoscurrin-2-like [*Dermacentor silvarum*]
trinity_SMG_TRINITY_DN148906_c0_g1_i1_1	648.1	1.97	8.36	6.94	4.56E-03	XP_037558824.1 shematrin-like protein 2 [*Dermacentor silvarum*]
trinity_SMG_TRINITY_DN9983_c0_g1_i1_1	344.35	1.14	8.17	6.03	1.52E-03	KAH7939644.1 hypothetical protein HPB52_015538 [*Rhipicephalus sanguineus*]
trinity_SMG_TRINITY_DN28987_c1_g2_i1_1	474.25	2.79	7.43	6.49	6.3E-03	XP_049268204.1 keratin-associated protein 21-1-like [*Rhipicephalus sanguineus*]
trinity_FM3_TRINITY_DN126090_c0_g1_i2_1	186.43	1.18	7.24	5.16	1.08E-03	XP_050031932.1 venom serine carboxypeptidase-like [*Dermacentor andersoni*]
trinity_SMG_TRINITY_DN34966_c0_g1_i12_1	214.74	1.46	7.17	5.36	1.73E-03	XP_037571958.1 acanthoscurrin-1-like [*Dermacentor silvarum*]
trinity_SMG_CL1162Contig3_1	537.79	4.39	6.98	6.68	0.01	XP_037564710.1 chorion class B protein L11-like [*Dermacentor silvarum*]
trinity_SMG_TRINITY_DN3200_c0_g2_i1_1	240.61	2.06	6.87	5.53	3.47E-03	XP_054918061.1 holotricin-3-like [*Dermacentor andersoni*]
trinity_SMG_TRINITY_DN5403_c0_g1_i7_1	170.46	1.55	6.75	5.04	1.95E-03	AAY66991.1 ribosomal protein L39 [*Ixodes scapularis*]
trinity_SMG_TRINITY_DN18337_c0_g1_i2_1	1,446.37	18.66	6.35	8.11	3.34E-02	XP_050050349.1 elastin-like [*Dermacentor andersoni*]
trinity_SMG_CL15407Contig1_1	1,033.86	19.15	5.83	7.63	3.63E-02	XP_037564729.1 acanthoscurrin-2 [*Dermacentor silvarum*]

In the midguts unfed male ticks, we observed a total of 263 DEGs, of which 159 up-regulated and 104 were down-regulated. In contrast, the unfed female tick midguts exhibited a shift with 529 DEGs identified. Of these, 288 were up-regulated, while 241 were down-regulated (see **Fig 4B and 4C**).

Key up-regulated genes in the midgut of unfed male ticks included a histone H3 protein, unigenes of histidine ammonia-lyase (3), unigenes involved in immune system of ticks were also up-regulated like hebreain (1) and microplusin like (1), lysozyme (2), mucin (1) and peritrophin (2) **Table 4** and **S1_Data.** In the midguts of unfed female ticks, we also observed the up-regulation of histone proteins (H3, H2A, H2B, H4 among others), known to play a role in gene expression and regulation. Additionally, a range of other protein associated with gene expression and regulation were up-regulated, including RNA binding protein (1), helicases (3), transcription (5) and translation (2) initiation and elongation factor proteins. There was also up-regulation of genes involved in immunity and oxidative stress response such amercin (1), glutathione peroxidase (3), cytochrome c oxidase (1) and peroxiredoxin (2) as well as a GDP-fucose protein O-fucosyltransferase (1). A detailed information of the DEGs in the unfed midguts of female *A*. *hebraeum* ticks compared to their corresponding non-infected control is provided in **Table 5** and **S1 Data**.

**Table 4:** Selected top DEGs up-regulated in *E*. *ruminantium* infected *A*. *hebraeum* unfed male midgut tissues.

Unigene ID	TPM_Pos	TPM_Neg	log2FC	logCPM	FDR	Blast_nr Annotation
trinity_MM2_TRINITY_DN50722_c0_g1_i9_1	1,570.25	4.4	8.84	9.90	0.017	XP_037279770.1 uncharacterized protein LOC119172726 [*Rhipicephalus microplus*]
trinity_SMG_TRINITY_DN6410_c0_g1_i17_1	818.44	2.99	8.44	8.96	0.013	XP_050022500.1 amine sulfotransferase-like [*Dermacentor andersoni*]
trinity_MM0_CL622Contig2_1	87.17	4.76	4.56	5.78	0.006	XP_050025843.1 G-patch domain and KOW motifs- -like isoform[*Dermacentor andersoni*]
trinity_FM3_TRINITY_DN1519_c1_g2_i1_1	31.99	2.26	4.15	4.41	0.007	XP_037502801.1 histidine ammonia-lyase [*Rhipicephalus sanguineus*]
trinity_SMG_TRINITY_DN16884_c0_g1_i2_1	16.89	1.29	3.98	3.59	0.001	AAR97292.1 hebreain [*Amblyomma hebraeum*]
trinity_FM3_TRINITY_DN18829_c0_g1_i3_1	39.07	3.29	3.92	4.69	0.007	DAA34703.1 TPA_inf: peritrophin [*Amblyomma variegatum*]
trinity_FM0_SCL12Contig234_1	8.78	1.02	3.34	2.88	0.005	XP_040358836.2 RNase H [*Ixodes scapularis*]
trinity_MM2_TRINITY_DN2773_c0_g1_i9_1	17.83	1.41	3.15	3.55	0.001	XP_049527598.1 LOW QUALITY PROTEIN: histone H3 [*Dermacentor silvarum*]
trinity_MM2_CL67766Contig1_1	21.89	3.48	3.01	4.00	0.011	DAA34703.1 TPA_inf: peritrophin [*Amblyomma variegatum*]
trinity_MM0_TRINITY_DN16908_c0_g1_i2_1	6.66	1.08	2.88	2.61	0.018	XP_050047877.1 fibronectin-like [*Dermacentor andersoni*]
trinity_MM0_CL1154Contig4_1	19.39	3.55	2.81	3.87	0.010	DAA34262.1 TPA_inf: hypothetical conserved protein 57, partial [*Amblyomma variegatum*]

**Table 5:** Selected top DEGs up-regulated in *E*. *ruminantium* infected *A*. *hebraeum* unfed female midgut tissues.

Unigene ID	TPM_Pos	TPM_Neg	log2FC	logCPM	FDR	Blast_nr Annotation
trinity_MM0_TRINITY_DN2641_c0_g1_i2_1	433.57	1.72	8.07	7.94	5.60E-03	XP_037285052.1 tigger transposable element-derived protein 6-like [*Rhipicephalus microplus*]
trinity_MM0_CL28891Contig1_1	345.96	1.45	7.97	7.61	3.13E-04	XP_049272112.1 GDP-fucose protein O-fucosyltransferase 2 isoform [*Rhipicephalus sanguineus*]
trinity_FM0_CL16750Contig1_1	1,787.61	14.41	7.14	9.98	2.88E-02	XP_050037664.1 sulfotransferase ssu-1-like [*Dermacentor andersoni*]
trinity_SMG_CL3467Contig1_1	156.13	1.68	6.63	6.49	8.55E-04	ACF35524.1 putative legumain-like protease precursor [*Dermacentor variabilis*]
trinity_FM0_SCL8Contig1934_1	172.23	8.07	4.57	6.67	9.60E-03	XP_037512849.1 DNA methyltransferase 1-associated protein 1 [*Rhipicephalus sanguineus*]
trinity_SMG_TRINITY_DN70050_c2_g1_i1_1	13.82	1.01	3.81	3.33	1.04E-03	XP_049527598.1 histone H3 [*Dermacentor silvarum*]
trinity_MM2_TRINITY_DN441587_c0_g1_i1_1	160.12	14.04	3.70	6.61	1.78E-02	XP_050029936.1 ubiquitin-like protein ATG12 [*Dermacentor andersoni*]
trinity_SMG_TRINITY_DN4083_c0_g1_i2_1	23.65	2.39	3.43	4.01	6.84E-03	KAH9365510.1 hypothetical protein HPB48_016288 [*Haemaphysalis longicornis*]
trinity_MM2_CL21973Contig1_1	12.82	1.57	3.12	3.29	3.99E-03	XP_037291604.1 histone H2A.V [*Rhipicephalus microplus*]
trinity_FM3_TRINITY_DN2480_c1_g1_i2_1	9.37	1.17	3.06	2.94	1.52E-03	XP_049527598.1histone H3 [*Dermacentor silvarum*]
trinity_MM0_SCL3Contig888_1	69.09	9.55	3.04	5.49	1.15E-02	XP_042143577.1 N-terminal kinase-like protein [*Ixodes scapularis*]
trinity_FM3_SCL32Contig104_1	17.52	2.34	3.03	3.67	7.22E-03	XP_037278994.1 N-alpha-acetyltransferase 50-like [*Rhipicephalus microplus*]

In the midgut tissues of partly-fed male ticks, we identified 303 DEGs. Among these, 170 genes showed increased expression (up-regulation), while 133 exhibited decreased expression (down-regulation) (**Fig 4D)**. On the other hand, in the midgut tissues of partly-fed female ticks, a total of 305 DEGs were identified. This group showed a higher proportion of up-regulation, with 205 genes demonstrating increased expression. In contrast, 100 genes were down-regulated (**Fig 4E)**. In terms of specific genes, partly-fed male ticks showed increased expression of multiple uncharacterized proteins, MAM and LDL-receptor class A, ixosin precursor, putative cement protein, among others (**Table 6)**. On the other hand, in the midgut tissues of partly fed female ticks, we observed a surge in the up-regulation of antimicrobial peptides including amercin (4) and holocin (1). There was also notable up-regulation of cytochrome P450 (8), inositol oxygenase (2), and tenascin R (2). Top annotated DEGs in this category are presented in **Table 7**, the complete set is detailed in **S1 Data.**

**Fig 5:**
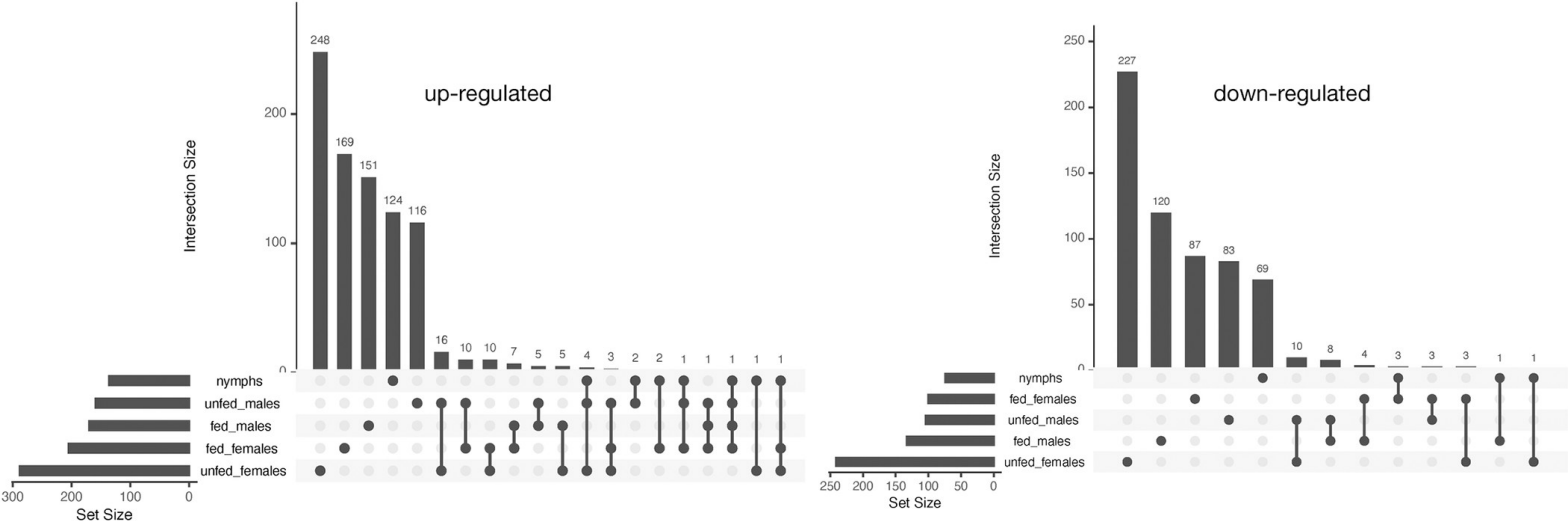
UpsetR plot of differential gene expression in *E*. *ruminantium* infected *A*. *hebraeum* midgut tissue. This figure illustrates the number of up-regulated and down-regulated DEGs common to the five midgut comparison groups. It highlights the unique and shared DEGs in nymphs, unfed and partly-fed male and female tick midguts.

Using UpSetR package, we identified common DEGs among the five midgut comparison groups. The midguts of unfed female ticks had the most unigenes that were exclusively up-regulated and down-regulated at 248 and 227 unigenes respectively. The same unfed tick groups had the highest commonly shared up-regulated (16) and down-regulated (10) unigenes compared to partly-fed group with seven up-regulated and four down-regulated (see **Fig 5**). Notable annotated unigenes that were common in unfed tick midgut groups include the histone H3, a DNA methyltransferase, membrane-associated tyrosine- and threonine-specific cdc2-inhibitory kinase-like, and elongation factor 1while in partly-fed group common up-upregulated include Kunitz protease inhibitor, cytochrome P450 and amercin. There was one uncharacterized unigene up-regulated in all the midgut groups except in the unfed female ticks. The number of DEGs commonly up-regulated and down-regulated among the five midgut groups are presented in **Fig 5**.

**Table 6:** Selected top DEGs up-regulated in *E*. *ruminantium* infected *A*. *hebraeum* partly-fed male midgut tissues.

Unigene ID	TPM_Pos	TPM_Neg	log2FC	logCPM	FDR	Blast_nr Annotation
trinity_MM0_CL40198Contig1_1	212.63	1.12	7.26	6.77	5.92E-03	XP_037521269.1 uncharacterized protein LOC119398274 [*Rhipicephalus sanguineus*]
trinity_SMG_CL4330Contig1_1	110.29	1.2	6.23	5.86	5.26E-03	XP_049268915.1 MAM and LDL-receptor class A [*Rhipicephalus sanguineus*]
trinity_MM2_TRINITY_DN8534_c0_g1_i1_1	224.13	9.33	4.41	6.90	1.75E-02	Q2LKX9.1 RecName: Full = Ixosin; Flags: Precursor [*Ixodes sinensis*]
trinity_MM2_SCL4Contig894_1	412.97	31.97	3.87	6.98	4.58E-02	XP_050043212.1 legumain-like [*Dermacentor andersoni*]
trinity_MM2_CL9006Contig1_1	32.15	2.45	3.49	4.31	9.37E-03	DAA34429.1 heme lipoprotein precursor, partial [*Amblyomma variegatum*]
trinity_MM2_TRINITY_DN396559_c0_g1_i1_1	25.9	2.26	3.29	4.05	8.55E-03	XP_037523995.1 heat shock protein HSP 90-alpha [*Rhipicephalus sanguineus*]
trinity_MM2_TRINITY_DN16778_c0_g1_i1_1	11.56	1.03	3.18	3.11	6.90E-03	XP_037269514.1 annexin-B12-like [*Rhipicephalus microplus*]
trinity_MM2_CL35167Contig1_1	22.97	2.32	3.08	3.92	7.89E-03	Q2LKX9.1 RecName: Full = Ixosin; Flags: Precursor [*Ixodes sinensis*]
trinity_MM0_TRINITY_DN1590_c0_g1_i1_1	43.51	4.99	2.93	4.75	1.56E-02	DAA34143.1 hypothetical secreted glycine-rich protein 11 [*Amblyomma variegatum*]
trinity_FM3_TRINITY_DN249947_c0_g1_i1_1	39.77	5.67	2.62	4.66	1.95E-02	XP_054926967.1 gamma-butyrobetaine dioxygenase-like [*Dermacentor andersoni*]
trinity_SMG_CL16167Contig1_1	14.49	2.15	2.53	3.42	6.08E-03	DAA34580.1 putative cement protein [*Amblyomma variegatum*]
trinity_MM2_TRINITY_DN22104_c0_g2_i1_1	9.72	1.58	2.37	2.99	2.51E-02	ACF35525.1 putative cathepsin B-like cysteine protease form 1, [*Dermacentor variabilis*]
trinity_MM2_SCL15Contig375_1	10.88	1.98	2.23	3.13	2.74E-02	XP_037523702.1 estradiol 17-beta-dehydrogenase 8 [*Rhipicephalus sanguineus*]
trinity_FM0_CL12207Contig1_1	8.54	1.56	2.21	2.87	3.898E-02	XP_037563560.1 steroid 17-alpha-hydroxylase/17,20 lyase [*Dermacentor silvarum*]

**Table 7:** Selected top DEGs up-regulated in *E*. *ruminantium* infected *A*. *hebraeum* partly-fed female midgut tissues.

Unigene ID	TPM_Pos	TPM_Neg	log2FC	logCPM	FDR	Blast_nr Annotation
trinity_FM3_TRINITY_DN267757_c2_g1_i1_1	210.86	1.01	7.93	7.00	4.75E-03	XP_050043212.1 legumain-like [*Dermacentor andersoni*]
trinity_SMG_TRINITY_DN2863_c0_g1_i9_1	1209.17	23.16	6.11	9.54	2.32E-02	ABS87356.1 lospin 4 [*Amblyomma americanum*]
trinity_MM0_CL34433Contig1_1	57.14	1.31	5.71	5.15	3.27E-03	XP_037574212.1 cytochrome P450 3A43-like [*Dermacentor silvarum*]
trinity_MM0_TRINITY_DN10177_c7_g1_i1_1	326.5	14.41	4.90	7.68	2.00E-03	ABI74752.1 amercin [*Amblyomma americanum*]
trinity_FM0_TRINITY_DN4810_c0_g1_i9_1	47.52	4.01	3.93	4.96	7.86E-03	XP_037573200.1 facilitated trehalose transporter Tret1-like [*Dermacentor silvarum*]
trinity_SMG_TRINITY_DN9277_c0_g1_i9_1	34.38	3.09	3.83	4.51	9.34E-03	XP_037524439.1 TNF receptor-associated factor 3 isoform X3 [*Rhipicephalus sanguineus*]
trinity_FM3_TRINITY_DN9043_c0_g1_i11_1	26.94	2.75	3.64	4.26	1.09E-02	XP_037527285.1 alpha-crystallin A chain [*Rhipicephalus sanguineus*]
trinity_SMG_TRINITY_DN20314_c0_g1_i1_1	9.54	1.21	3.25	2.98	3.77E-03	QEO24728.1 holosin 4 [*Ixodes holocyclus*]
trinity_MM0_TRINITY_DN107829_c0_g1_i1_1	10.44	1.35	3.23	3.08	2.62E-03	ABI74752.1 amercin [*Amblyomma americanum*]
trinity_MM2_TRINITY_DN1389_c1_g1_i17_1	12.68	1.89	3.06	3.32	8.19E-04	ABI74752.1 amercin [*Amblyomma americanum*]
trinity_MM2_CL76749Contig1_1	26.53	4.17	3.04	4.25	1.67E-03	XP_037582082.1 tenascin-R isoform X1 [*Dermacentor silvarum*]
trinity_MM0_TRINITY_DN15074_c0_g1_i9_1	46.57	7.57	3.01	5.02	1.77E-02	XP_037570085.1 MAM and LDL-receptor class A domain like [*Dermacentor silvarum*]
trinity_SMG_TRINITY_DN8568_c3_g1_i2_1	9.15	1.4	3.00	2.96	7.21E-03	XP_050040171.1 cytochrome P450 3A14-like [*Dermacentor andersoni*]

### Functional enrichment analysis of DEGs

**Fig 6:**
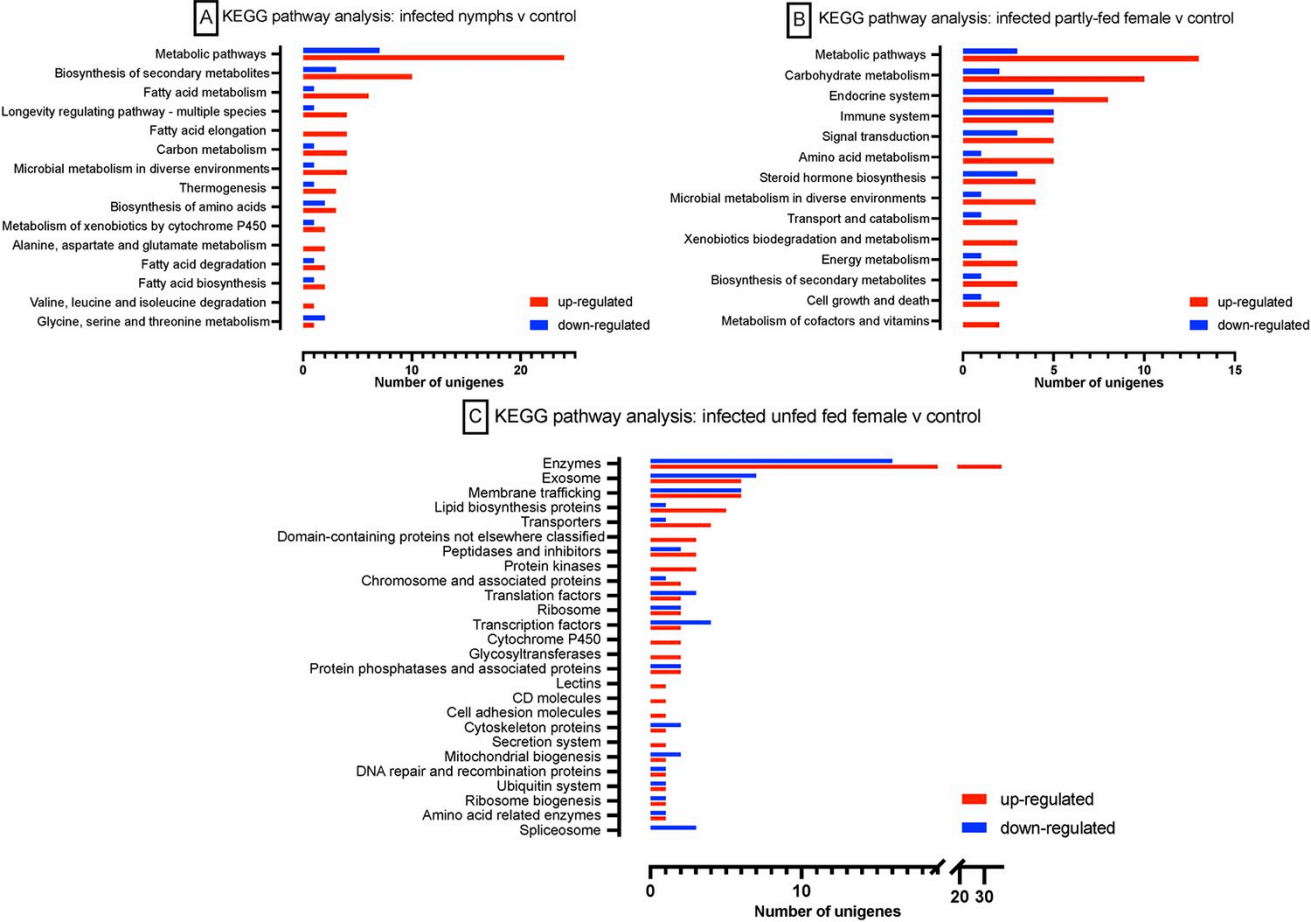
KEGG pathway analysis of selected categories of *A*. *hebraeum* midgut transcriptional response to *E*. *ruminantium* infection. Panel [A] depicts the comparative analysis of DEGs in the midguts of infected nymphs against uninfected controls. Panel [B] elucidates the DEG patterns in infected partly-fed females compared to their respective controls and [C] infected unfed females compared to control.

KEGG pathway analysis was performed to ascertain the probable biological pathway of DEGs in various categories. In the *E*. *ruminantium* infected *A*. *hebraeum* midguts, we observed changes in the gene expression patterns pertaining to several key biological pathways. In general, metabolic pathways and biosynthesis of secondary metabolites were up-regulated across infected midgut categories compared to the control ticks (see **Fig 6**).There was also a general up-regulation of unigenes involved in fatty acid biosynthesis, elongation, and degradation as well as in amino acid metabolism, the genes involved in alanine, aspartate and glutamate metabolism, and glycine, serine and threonine metabolism were up-regulated in the infected group. In infected, unfed ticks, we observed a pronounced up-regulation of genes related to chromosomes and associated proteins, as well as those involved in the activities of glycosyltransferases and protein kinases (**Fig 6C**) which could suggest an increased demand for cellular processes, such as DNA replication and repair, protein modification, and signal transduction. Key KEGG pathways affected in *E*. *ruminantium* infection in the midgut of nymphs, unfed and partly-fed females is illustrated in **Fig 6.**

### qPCR experimental validation

**Fig 7:**
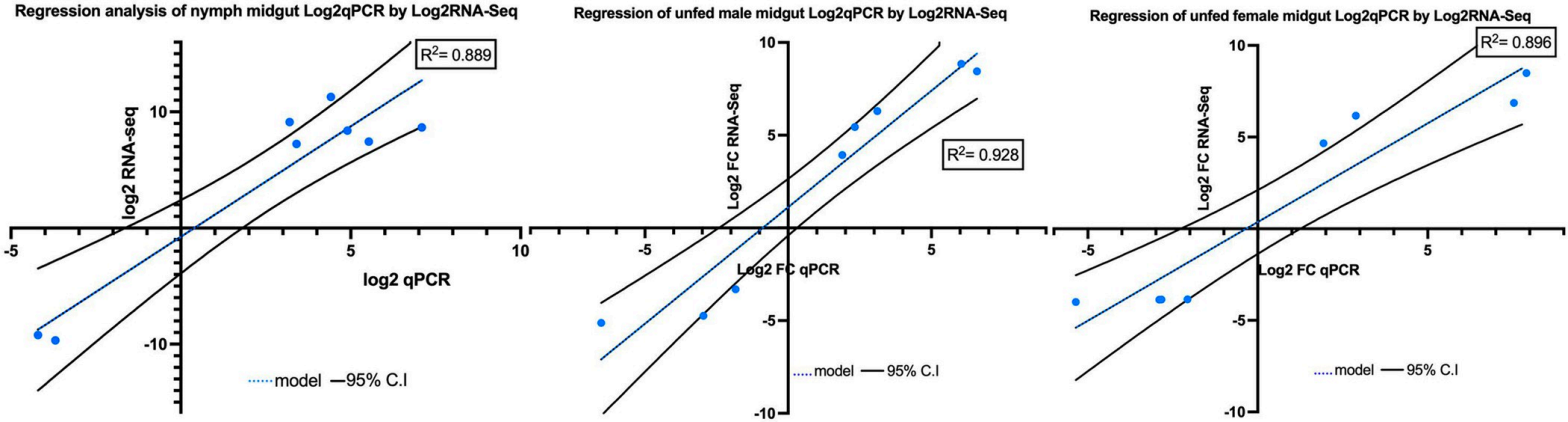
Selected regression plots comparing the differential expression values of eight genes in *A*. *hebraeum* nymphs, and the unfed male and female tick midguts. This comparison is between the quantitative real-time PCR (qPCR) data and the RNA-seq data. The log2 fold-change values from qPCR are plotted on the x-axis and the corresponding values from RNA-Seq are plotted on the y-axis. The plot illustrates the correlation and consistency between the qPCR validation and the original RNA-Seq data for these selected genes. All validation plots are detailed in **S2 Data**.

To validate our RNA-seq analysis results, we analyzed 40 DEGs, eight for each of the five midgut categories using qPCR. Selected DEGs in nymphs, unfed male and female tick midguts are presented in **Table 8.** The *A*. *hebraeum* translation elongation factor 1 alfa was used as a reference gene. The expression patterns of the DEGs obtained using qRT-PCR were largely congruent with the RNA-seq results with slight variations, indicating that the RNA-seq results could be reliably used to further infer the expression of other genes involved in *A*. *hebraeum–E*. *ruminantium* interaction (**Fig 7).**

**Table 8:** Selected DEGs values of 8 genes of *A*. *hebraeum* unfed male midgut. The log2 ratios qPCR plotted against those of respective RNA-Seq.

	Unigene ID	Annotation	Fold change	log2qPCR	Fold change	log2RNA-seq
Nymph	trinity_SMG_CL5318Contig2_1	acanthoscurrin-like	680.28	9.41	2,486.67	11.28
	trinity_SMG_CL77607Contig1_1	uncharacterized protein	9.18	3.2	556.41	9.12
	trinity_SMG_CL233Contig5_1	acanthoscurrin-2-like	136.23	7.09	404.50	8.66
	trinity_SMG_TRINITY_DN148906_c0_g1_i1_1	shematrin-like protein 2	29.85	4.9	333.14	8.38
	trinity_SMG_TRINITY_DN28987_c1_g2_i1_1	keratin-associated protein	46.21	5.53	172.44	7.43
	trinity_FM3_TRINITY_DN126090_c0_g1_i2_1	venom serine carboxypeptidase	10.62	3.41	151.16	7.24
	trinity_MM0_TRINITY_DN34998_c0_g1_i4_1	Unknown	0.054	-4.21	0.0016	-9.22
	trinity_FM3_CL75372Contig1_1	Unknown	0.077	-3.69	0.0012	-9.68
Unfed males	trinity_MM0_TRINITY_DN2641_c0_g1_i1_1	unknown	238.85	7.9	359.53	8.49
	trinity_SMG_CL34206Contig1_1	putative legumain-like precursor	184.82	7.53	116.16	6.86
	trinity_SMG_TRINITY_DN20196_c0_g2_i2_1	histone H3	7.36	2.88	71.51	6.16
	trinity_SMG_TRINITY_DN2863_c0_g1_i9_1	amercin	3.81	1.93	25.28	4.66
	trinity_FM3_TRINITY_DN2009_c0_g1_i10_1	inositol oxygenase	0.239	-2.06	0.069	-3.85
	trinity_FM0_SCL2340Contig1_1	TNF receptor-associated factor 6	0.140	-2.83	0.068	-3.87
	trinity_MM2_CL956Contig2_1	tenascin-R	0.134	-2.89	0.068	-3.87
	trinity_MM0_TRINITY_DN34998_c0_g1_i4_1	unknown	0.024	-5.35	0.062	-3.99
Unfed females	trinity_MM2_TRINITY_DN50722_c0_g1_i9_1	unknown	65.79	6.04	458.25	8.84
	trinity_SMG_TRINITY_DN6410_c0_g1_i17_1	unknown	96.33	6.59	347.29	8.44
	trinity_SMG_TRINITY_DN20196_c0_g2_i2_1	histone H3	8.63	3.11	78.24	6.29
	trinity_FM0_TRINITY_DN13079_c0_g1_i1_1	hebreain	4.99	2.32	43.41	5.44
	trinity_FM3_TRINITY_DN18829_c0_g1_i3_1	Reverse transcriptase	3.71	1.89	15.13	3.92
	trinity_FM3_SCL11Contig604_1	hypothetical protein HPB47_024567	0.281	-1.83	0.100	-3.31
	trinity_MM0_TRINITY_DN131493_c0_g1_i1_1	unknown	0.129	-2.95	0.037	-4.74
	trinity_FM0_TRINITY_DN87676_c0_g1_i2_1	unknown	0.011	-6.52	0.028	-5.12

## Discussion

In this study, we analyzed the midgut tissues of *A*. *hebraeum* ticks following successful acquisition of *E*. *ruminantium* by the nymphs. However, the emerging adult ticks were unable to transmit the pathogen to naïve sheep in subsequent experiments. The reasons for this remain unclear, but the dosage of the pathogen could potentially play a role. During the acquisition experiment, we were able to control the inoculated dosage in sheep. In the transmission experiment, we sought to replicate the natural transmission dynamics of *E*. *ruminantium* as they occur in the field. Consequently, the pathogen dosage likely administered by the ticks may have been too low, which may have contributed to the lack of successful transmission observed in this study. This finding underscores the complex interplay between vector, pathogen, and host that must be better understood in order to effectively manage ticks and tick-borne diseases. We observed substantial differences in *E*. *ruminantium* read counts among infected ticks, with midguts of unfed infected adults having higher counts than partly-fed adults and nymphs **Table 1**. This could be suggestive of higher *E*. *ruminantium* load in the unfed adults compared to partly-fed counterparts. The difference could be attributed to a possible migration of the pathogen from midgut to SG or other organs as has been previously demonstrated for *Borrelia* species that remain in the gut and migrate to the SG and other organs during the next blood meal [52].

Using Illumina short-read sequencing, we have delineated potentially important tick genes in *A*. *hebraeum* that have been reported in other tick-pathogen models. These include specific genes that appear to play a significant role in the defense against, or facilitation of, *E*. *ruminantium* entry into the tick host. Using RNA-seq of a single replicate of the three prepared for each treatment followed by RT-qPCR validation of all three biological replicates, we show differences in the expression of genes between ticks that had acquired *E*. *ruminantium* and their corresponding controls. Our approach not to sequence all the replicates has potential limitations as variation, errors, and biases is higher on analysis of single replicate compared to three [53]. Though a number of normalization steps have been developed for NGS data to remove unwanted variance [54], sequencing three biological replicates may have improved the quality of data presented. Previous transcriptomic studies described *E*. *ruminantium* DEGs induced during infection in *vitro* and SG [29,55] or used alternative approaches for host gene identification [56].

A total of 102,036 unigenes were assembled and using BLAST-NR database with an E-value threshold of 1E-05, we were able to annotate 55,581 of these, accounting to ~55% of the total. The majority of these annotations were related to tick sequences. However, when we included a query coverage threshold of 50%, the number of successfully annotated unigenes dropped drastically to only 21% of the total. This indicates that many of the sequences in our assembly were partial or have less homology to the reference sequences, leading to lower coverage in BLAST alignment. Hence, to ensure a more comprehensive annotation of our data, we chose not to include a stringent query coverage threshold. The remaining 46,455 unigenes, making up 45.52% of the total, could not be functionally annotated and are currently classified as unknown. Within this group of unannotated unigenes, we identified 4,673 that had a match with the Gulf Coast tick, *Amblyomma maculatum*. However, it should be noted that the full set of protein sequences from the *A*. *maculatum* draft genome project, is not yet available in public repositories, limiting our ability to fully annotate these matching unigenes [57]. Our successfully annotated unigenes (55.58%) are marginally higher than 30.56% and 32.14% previously reported in the *de novo* assembly and annotation of *Haemaphysalis flava* and the red poultry mite *Dermanyssus gallinae* respectively [58,59]. In the top-hit species distribution of *A*. *hebraeum* unigenes, the highest percentage of unigenes aligned to the Taiga tick *D*. *silvarum* (24.73%), followed by those that aligned with the brown dog tick *R*. *sanguineus* (18.92%) and only 1.59% mapped to *A*. *variegatum* which is closely related to *A*. *hebraeum*. The successful annotation of a relatively high proportion of our unigenes was greatly facilitated by the availability of existing tick genomic resources, particularly those developed in a large-scale comparative study of tick genomes and understanding of their genetic diversity [60].

Although the midgut transcriptomes of several tick species have been previously described in efforts to identify DEGs involved in blood meal digestion and other physiological processes [28,31,61,62], transcriptome studies investigating the role of midgut DEGs that act as barriers or gateway to pathogen invasion are scant. In our analysis, we noted a number of genes in the infected midgut which had been previously reported to be involved in host-pathogen interactions and immunity [63,64]. For example, in our nymph midgut category, we observed an increase in expression of both antimicrobial peptides and lectins in the *E*. *ruminantium*-infected nymph midgut compared to its corresponding non-infected control. There was a significant up-regulation of two isoforms of the acanthoscurrin protein as a result of the infection. Acanthoscurrin is an antimicrobial agent characterized by its unique glycine-rich structure. Initially identified in the hemocytes of the spider *Acanthoscurria gomesiana*, this protein has shown efficacy against *Candida albicans* and *E*. *coli* [65]. Acanthoscurrin like protein has also recently been observed to be up-regulated in response to acquisition *B*. *burgdorferi* by juvenile *I*. *pacificus* ticks, cementing its role in protecting the host from invasion by pathogens [66]. We also observed notable up-regulation of galectin-4-like and techylectin-5A. Techylectin is a fibrinogen related protein that has been reported to be important in the agglutination of both Gram negative and Gram positive bacteria [67] and galectin-4 is glycoproteins characterized by a carbohydrate recognition domain implicated in translocation and cellular trafficking of protein across epithelial cells [68]. PpGalec in particular is a tandem repeat galectin expressed in the midgut of the sand fly *Phlebotomus papatasi* and was shown to mediate *Leishmania major-*specific binding to the insect midgut, an event crucial for parasite survival and accounts for species-specific vector competence [69].

In both infected unfed male and female ticks’ midguts, a significant up-regulation in the expression of histone proteins was observed. This finding correlates with earlier research that highlighted the ability of *A*. *phagocytophilum* to modify the histone proteins of *I*. *scapularis* [70]. This alteration was shown to hamper programmed cell death in order to permit the pathogens survival and proliferation within the host [71]. Given these factors, it is conceivable that *E*. *ruminantium* could employs similar tactics during its infection cycle in tick cells, which might allow it to evade the tick’s immune responses. GDP-fucose protein O-fucosyltransferase 2 was up-regulated in the *E*. *ruminantium* infected midguts of unfed female ticks, but there was no observation of fucosyltransferase in the unfed males or partly-fed male or female tick midguts. Fucosyltransferase is an enzyme that adds fucose sugar to proteins and fucosylation activity has been linked as a hard tick factor important in pathogen transmission [72]. Previous studies have reported that *A*. *phagocytophilum* modulates the expression of α-1,3-fucosyltransferase during the acquisition phase in *I*. *scapularis* midgut and gene silencing significantly reduced the pathogen colonization of both tick midgut cells and murine mast cells *in vitro* [73,74]. Moreover, α-1,3-fucosyltransferase has been demonstrated to significantly enhance the susceptibility of *D*. *andersoni* midgut cells to *Anaplasma marginale* infection *in vitro* [75].

Our analyses revealed other tick immunity genes that were up-regulated in the *E*. *ruminantium* infected partly-fed female or male tick midguts. Two particular proteins of interest were amercin that was up regulated in both partly-fed groups and holocin-4 that was up-regulated in partly-fed females.

Holocin is an antimicrobial agent that has been shown to abrogate the proliferation *Staphylococcus aureus* and *Fusarium graminearum* in the *Ixodes holocyclus* tick species [76]. Amercin is an immunity protein originally found to be predominantly synthesized in the midgut, fat bodies, and salivary glands of the Lone Star tick, *A*. *americanum* [77]. We observed four unigenes of amercin that up-regulated in partly-fed female tick midgut and one unigene in the male groups (**S1 Data**). This could suggest its role in shielding both sexes from *E*. *ruminantium* incursions.

Our investigation also led to the identification of other crucial unigenes up-regulated in the *E*. *ruminantium* infected midguts. These included antioxidant enzymes such as glutathione peroxidase (unfed females), glutathione S transferase (nymphs) and serine protease inhibitors (nymphs, and partly-fed groups) (**S1 Data**). Additionally, mucin unigenes (up-regulated in nymphs) and peritrophin unigenes (female midgut groups) were also up-regulated in the midgut of infected *A*. *hebraeum*. It has previously been postulated that these genes function in shaping gut homeostasis and are essential in mucosal immunity of *Anopheles gambiae* [78] and *I*. *scapularis* [79,80]. Serine proteases and protease inhibitors (Kunitz) have been shown modulate immune cascades involved in pathogen recognition and control [81]. Mucin and peritrophin are important innate immunity barriers with a direct impact on pathogen colonization of the midgut [82,83]. Serine protease inhibitors have been investigated in several recent studies focused on uncovering their role in the regulation of inflammation and complement activation in mammals [84]. They play a critical role in the immunity and physiology of arthropods [85] as illustrated by the unveiling of the differential expression of 45 serine protease inhibitors genes in the midgut and salivary glands of unfed and partly-fed *I*. *scapularis* ticks [86].

The KEGG pathway analysis has shade light into the alteration of gene expression pathways of five groups of *E*. *ruminantium* infected *A*. *hebraeum* tick midguts. We observed an up-regulation of genes related to the global metabolic pathways and biosynthesis of secondary metabolites that is suggestive of enhanced requirement for metabolic processes and secondary metabolites, which are typically instrumental in the defense mechanisms [87]. Fatty acid metabolism, including its biosynthesis, elongation, and degradation pathways, and amino acid metabolic pathways also showed an up-regulation, including those for alanine, aspartate, glutamate, glycine, serine, and threonine. The specific up-regulation of genes related to chromosomes and associated proteins, as well as those involved in the activities of glycosyltransferases and protein kinases in unfed female tick (**Fig 6C**) points a possible *E*. *ruminantium* mechanisms to adapt to unfed ticks. It may also indicate a heightened immune response, as glycosyltransferases and protein kinases are often implicated in pathogen recognition [88,89]. Collectively, provides an understanding the molecular mechanisms that regulate *A*. *hebraeum* immunity and interactions with *E*. *ruminantium*. We have pinpointed key genes that either prevent *E*. *ruminantium* from invading the midgut or potentially facilitate its entry. These findings not only shed light on the complex host-pathogen interplay occurring in the tick midgut, but also present promising targets for further investigations.

## Conclusion

In this study, we have used comparative transcriptomics to shed light on the complex interaction between the tick *A*. *hebraeum* and the pathogen *E*. *ruminantium* in the tick midgut. We successfully sequenced, assembled, and annotated 102,036 unigenes from *A*. *hebraeum* midgut tissues. By comparing infected and non-infected transcriptomes, we identified more than 2,000 DEGs, which are potentially key to the pathogen’s lifecycle within the midgut. This unprecedented insight into the host-pathogen interaction at a molecular level presents valuable targets for further investigation to develop effective anti-tick strategies or transmission-blocking vaccines, which could potentially revolutionize the control of tick-borne diseases like heartwater disease.

## Supporting information

S1 Table Tables of primers used for quantitative PCR (qPCR). (DOCX)

S2 Table Tables of *de novo* assembly statistics, alignment and functional annotations of *A*.*hebraeum* unigenes. (DOCX)

S1 Fig Fig of Gene Ontology and Cluster of Orthologous groups of *A*. *hebraeum* unigenes. (TIFF)

S1 Data Data on DEGs of five comparison groups of *E*. *ruminantium* infected *A*.*hebraeum* midguts compared to controls. (XLSX)

S2 Data Data of complete RNA-seq data validation using qPCR. (DOCX)
