# Supplementary material for: De novo assembly and annotation of the Amblyomma hebraeum tick midgut transcriptome response to Ehrlichia ruminantium infection
Source: PLoS Negl Trop Dis. 2023 Aug 14;17(8):e0011554. doi: 10.1371/journal.pntd.0011554 (PMC10449191; doi:10.1371/journal.pntd.0011554)
Supplement: S1 Table — (DOCX) [file pntd.0011554.s001.docx]

Supplementary Material

*De novo* assembly and annotation of the *Amblyomma hebraeum* tick midgut transcriptome response to *Ehrlichia ruminantium* infection

David Omondi^*^, Erich Zweygarth, Edwin Murungi, Frans Jongejan, Ard M. Nijhof

*** Correspondence:** David Omondi: [domolbio@gmail.com](mailto:domolbio@gmail.com)

Table A in S1_Table of primer list used for *Amblyomma hebraeum* nymph midgut transcriptome validation

| **Unigene ID** | **Blast_nr Annotation** | **Forward primer** | **Reverse primer** | **size** |
| --- | --- | --- | --- | --- |
| trinity_SMG_CL5318Contig2_1 | XP_037268604.1 acanthoscurrin-2-like [*R. microplus*] | GGCTATGGTGGCTATGGCTT | GCCACGGCTTTTGAGAATCC | 176 |
| trinity_SMG_CL77607Contig1_1 | XP_054934363.1 uncharacterized protein LOC126543383 [*D. andersoni*] | CTTTCGAAATCTCGCTGGCG | TTGGGAAAGTGGCTGTGTGT | 185 |
| trinity_SMG_CL233Contig5_1 | XP_037564726.1 acanthoscurrin-2-like [*D. silvarum*] | GTTCCTCCATCGACACGGTT | TATTTCAACAGGAGCGCCGT | 170 |
| trinity_SMG_TRINITY_DN148906_c0_g1_i1_1 | XP_037558824.1 shematrin-like protein 2 [*D. silvarum*] | AGCTGCTGTCTACCGAGGTA | ACATGAAGGTAACGGCGTGT | 154 |
| trinity_SMG_TRINITY_DN28987_c1_g2_i1_1 | XP_049268204.1 keratin-associated protein 21-1-like [*R. sanguineus*] | CTTCACGACTCCAACTCCCC | TAGCTTGGCATCGCTCGTTT | 151 |
| trinity_FM3_TRINITY_DN126090_c0_g1_i2_1 | XP_050031932.1 venom serine carboxypeptidase-like [*D. andersoni*] | ACCGGAAGCCACTTGTTCTT | AGTGGGTTCGACTGCGTTAG | 186 |
| trinity_MM0_TRINITY_DN34998_c0_g1_i4_1 | Unknown | TTTCTTAGGGGTGTGCGCTT | TTACGGCCACACCCAAGTTT | 181 |
| trinity_FM3_CL75372Contig1_1 | Unknown | GGGTCTAGAGGATGGGCTGA | CTGTCCCACTACTGCGACAC | 197 |

| Table B in S1_Table of primer list used for *Amblyomma hebraeum* unfed female midgut transcriptome validation | | | | |
| --- | --- | --- | --- | --- |
| **Unigene ID** | **Blast_nr Annotation** | **Forward primer** | **Reverse primer** | **size** |
| trinity_MM0_TRINITY_DN2641_c0_g1_i1_1 | unknown | GTATTGACTCTGCAAGCGCG | ATCCCTTTCAGCGCAGACAA | 200 |
| trinity_SMG_CL34206Contig1_1 | ACF35524.1 putative legumain-like protease precursor [*D. variabilis*] | CACTGGGCACTTCTTGTTGC | GATGATCACTCCAGGCCTCG | 177 |
| trinity_SMG_TRINITY_DN20196_c0_g2_i2_1 | XP_049527598.1 LOW QUALITY PROTEIN: histone H3 [*D. silvarum*] | TTTTACACGCGTCGCCAAAG | CACATGTCTGCTTTGGCCAC | 191 |
| trinity_SMG_TRINITY_DN2863_c0_g1_i9_1 | ABI74752.1 amercin [*A. americanum*] | CTTTGGCTGCCCGTTTAACC | GCGAATACCATCAGGAGCGA | 158 |
| trinity_FM3_TRINITY_DN2009_c0_g1_i10_1 | XP_050027273.1 inositol oxygenase-like [*D. andersoni*] | ATGGATACTGCATGCCGAGG | CGCAACCTCGTGTTTGTCAG | 157 |
| trinity_FM0_SCL2340Contig1_1 | XP_037526299.1 TNF receptor-associated factor 6 [*R. sanguineus*] | AGAACGAACACGCTCGAAGT | GATAAGGGTGCGGAGAGAGC | 180 |
| trinity_MM2_CL956Contig2_1 | XP_049512374.1 tenascin-R isoform X2 [*D. silvarum*] | CACAGGATTTCCCATGCCCT | TGCACGTTTCCGCTCTATGT | 150 |
| trinity_MM0_TRINITY_DN34998_c0_g1_i4_1 | unknown | TTTCTTAGGGGTGTGCGCTT | TTACGGCCACACCCAAGTTT | 181 |

Table C in S1_Table of primer list used for *Amblyomma hebraeum* unfed male midgut transcriptome validation

| **Unigene ID** | **Blast_nr Annotation** | **Forward primer** | **Reverse primer** | **size** |
| --- | --- | --- | --- | --- |
| trinity_MM2_TRINITY_DN50722_c0_g1_i9_1 | unknown | CCACCGGATCACTATGACCG | GGTGGGTAGTTTGACTGGGG | 168 |
| trinity_SMG_TRINITY_DN6410_c0_g1_i17_1 | unknown | TTTCCGGTGTAGCGGTGAAA | AGCTGCGAAACCGAAAGAGA | 185 |
| trinity_SMG_TRINITY_DN20196_c0_g2_i2_1 | XP_049527598.1 histone H3 [*D. silvarum*] | TTTTACACGCGTCGCCAAAG | CACATGTCTGCTTTGGCCAC | 191 |
| trinity_FM0_TRINITY_DN13079_c0_g1_i1_1 | AAR97292.1 hebreain [*A. hebraeum*] | AGAAGAACGACCAGGTGCTG | GTACTTGGCCATTGCTCCCT | 173 |
| trinity_FM3_TRINITY_DN18829_c0_g1_i3_1 | SCV66484.1 Reverse transcriptase (RNA-dependent DNA polymerase) | GTCCTGAACCTGATGCAGCT | CCGAAAGAGGCACAAAAGGC | 161 |
| trinity_FM3_SCL11Contig604_1 | KAG0428456.1 hypothetical protein HPB47_024567 [*I. persulcatus*] | TCTTCGTTGATGCTTCCGCT | GCCCCTGTGCACAAAAACAA | 156 |
| trinity_MM0_TRINITY_DN131493_c0_g1_i1_1 | unknown | GCCAAAGAAGAAGCGAAGGC | GGCAGAGTCAAAAGCACAGC | 180 |
| trinity_FM0_TRINITY_DN87676_c0_g1_i2_1 | unknown | GTAGGTAAGCCGCCGAGTAC | ATGGGAGCGGTTCGTCATTT | 166 |

Table D in S1_Table of primer list used for *Amblyomma hebraeum* fed female midgut transcriptome validation

| **Unigene ID** | **Blast_nr Annotation** | **Forward primer** | **Reverse primer** | **size** |
| --- | --- | --- | --- | --- |
| trinity_FM3_TRINITY_DN71869_c0_g3_i1_1 | unknown | ACATGTCCGGACTCATCTGC | TTTCTTAGGGGTGTGCGCTT | 151 |
| trinity_SMG_TRINITY_DN19612_c2_g4_i1_1 | KAH7939345.1 hypothetical protein HPB52_011352 [*R. sanguineus*] | TCACTCAGTCTCCTGCCTCA | TGGAGTGCCAGTCTACCTGA | 150 |
| trinity_SMG_TRINITY_DN2863_c0_g1_i9_1 | ABI74752.1 amercin [Amblyomma americanum] | CTTTGGCTGCCCGTTTAACC | GCGAATACCATCAGGAGCGA | 158 |
| trinity_FM3_TRINITY_DN3711_c0_g1_i8_1 | XP_050045023.1 steroid 17-alpha-hydroxylase/17,20 lyase-like [*D. andersoni*] | AGTCGCTCCTCTCATCCCTT | GCAGAGTGAGAAGGTTCCCC | 153 |
| trinity_SMG_TRINITY_DN839_c0_g1_i14_1 | XP_037562278.1 uncharacterized protein LOC119441739 [*D. silvarum*] | ATCGTCTCGCAGCTGCATTA | AACAGCGTCGTCACTAAGCA | 171 |
| trinity_FM3_TRINITY_DN4923_c3_g1_i1_1 | ABI74752.1 amercin [*A. americanum*] | GCCCATGCAAGCCCTTTTAC | GCGGCTTAAAATGCGTGTCA | 152 |
| trinity_FM3_TRINITY_DN116440_c0_g1_i2_1 | DAA34636.1 TPA_inf: hypothetical secreted protein 1496 [*A. variegatum*] | TGGGAAATTCCGTACGTGCA | TCGCACTTGCACAGAGATGT | 155 |
| trinity_MM2_TRINITY_DN307197_c0_g1_i1_1 | XP_050036064.1 neuropeptide-like protein 31 [*D. andersoni*] | GATAACCTCCGTACGCAGGG | CTACGGCCTCAGCGGATATG | 164 |
| trinity_FM0_TRINITY_DN21546_c0_g1_i1_1 | DAA34694.1 TPA_inf: peritrophin [*A. variegatum*] | ATTCAAGCCAGCAGACGACA | CAAATCGGAGACAGAGGCGT | 199 |

Table E in S1_Table of primer list used for *Amblyomma hebraeum* fed male midgut transcriptome validation

| **Unigene ID** | **Blast_nr Annotation** | **Forward primer** | **Forward primer** | **Size** |
| --- | --- | --- | --- | --- |
| trinity_MM0_CL40198Contig1_1 | unknown | ACGGTTCAGGATCGCATCTC | CCGGCTCTCTTAGTGGTGTG | 184 |
| trinity_SMG_CL4330Contig1_1 | XP_054932033.1 uncharacterized protein LOC126540486 [*D. andersoni]* | CTTGGTTTGTGCAGAGTGCC | TCCTCCTGCGTTTCCTTGTC | 158 |
| trinity_MM2_TRINITY_DN17428_c0_g1_i2_1 | XP_037284856.1 uncharacterized protein LOC119177773 iso X4 [*R. microplus*] | ACGCCCGATCTGTGAGAATC | ACGAGTCTTCAAGGCCTTGG | 158 |
| trinity_FM3_TRINITY_DN22490_c0_g1_i1_1 | XP_050043259.2 uncharacterized protein LOC126540484 [*D. andersoni*] | CGGCCAACATTCTCCAGACT | AAGAGCCACGTTCAGCTTCA | 177 |
| trinity_MM2_TRINITY_DN56378_c0_g1_i1_1 | XP_050047744.2 uncharacterized protein LOC126544455 [*D. andersoni*] | CTCAGGGCCTTTCGGGTTAC | CGGCTGTAAACAGTGAACGC | 200 |
| trinity_MM2_TRINITY_DN3_c12_g1_i1_1 | unknown | GTTCGGGCTCACTAACTCGG | GAGGTGGTGGGTTCGACTC | 156 |
| trinity_SMG_TRINITY_DN12092_c3_g1_i1_1 | unknown | CGTCACTGTGGGCTTGATGA | TGTCCCCCGTCTAGACACAT | 151 |
| trinity_SMG_CL8950Contig1_1 | unknown | GTACGAACGCCCAAGCTACT | AAAACGGTGAGCGCAGATTG | 168 |
