# Supplementary material for: De novo assembly and annotation of the Amblyomma hebraeum tick midgut transcriptome response to Ehrlichia ruminantium infection
Source: PLoS Negl Trop Dis. 2023 Aug 14;17(8):e0011554. doi: 10.1371/journal.pntd.0011554 (PMC10449191; doi:10.1371/journal.pntd.0011554)
Supplement: S2 Table — (DOCX) [file pntd.0011554.s002.docx]

Supplementary Material

*De novo* assembly and annotation of the *Amblyomma hebraeum* tick midgut transcriptome response to *Ehrlichia ruminantium* infection

David Omondi^*^, Erich Zweygarth, Edwin Murungi, Frans Jongejan, Ard M. Nijhof

*** Correspondence:** David Omondi: [domolbio@gmail.com](mailto:domolbio@gmail.com)

| \| Table A in S2_*A. hebraeum* midgut *de novo* assembly statistics \| \| \| \| --- \| --- \| --- \| \| **Type** \| **Transcript** \| **Unigene** \| \| Total number \| 571,913 \| 102,036 \| \| Total base \| 809,870,858 \| 210,526,621 \| \| Maximum length (bp) \| 145,921 \| 145,921 \| \| Minimum length (bp) \| 206 \| 211 \| \| Average length (bp) \| 1,639 \| 2,056 \| \| N50 length (bp) \| 3,196 \| 3,815 \| \| L50 length (bp) \| 84,718 \| 16,385 \|   Table B in S2_Indexed unigenes read back mapping statistics summary of *A.hebraeum* midgut reads | | | | | |
| --- | --- | --- | --- | --- | --- | --- | --- | --- | --- | --- | --- | --- | --- | --- | --- | --- | --- | --- | --- | --- | --- | --- | --- | --- | --- | --- | --- | --- | --- | --- | --- | --- |
| **Sample** | **Read_1** | **Read_2** | **Paired** | **Mapped** | **properly paired** |
| Nymph pos | 46,679,800 | 46,679,800 | 93,359,600 | 85.54% | 79.65% |
| Nymph neg | 47,319,799 | 47,319,799 | 94,639,598 | 83.43% | 76.78% |
| Unfed male pos | 44,281,921 | 44,281,921 | 88,563,842 | 77.91% | 71.40% |
| Unfed male neg | 47,539,555 | 47,539,555 | 95,079,110 | 78.86% | 72.11% |
| Day-2-fed male pos | 41,478,216 | 41,478,216 | 82,956,432 | 82.67% | 77.07% |
| Day-2-fed male neg | 41,833,626 | 41,833,626 | 83,667,252 | 82.44% | 76.91% |
| Unfed female pos | 47,585,496 | 47,585,496 | 95,170,992 | 80.56% | 74.29% |
| Unfed female neg | 45,680,511 | 45,680,511 | 91,361,022 | 78.93% | 72.10% |
| Day-3-fed female pos | 60,423,710 | 60,423,710 | 120,847,420 | 82.70% | 76.34% |
| Day-3-fed female neg | 41,012,690 | 41,012,690 | 82,025,380 | 82.81% | 76.35% |

| Table C in S2_Functional annotations of unigenes of *A. hebraeum* | | |
| --- | --- | --- |
| **Database** | **Unigenes** | **Percentage (%)** |
| Annotated in NR | 54,080 | 53.01 |
| Annotated in KO | 15,463 | 15.15 |
| Annotated in SwissProt | 23,090 | 22.62 |
| Annotated in Pfam | 5,135 | 5.03 |
| Annotated in GO | 3,294 | 3.22 |
| Annotated in KOG | 8,656 | 8.48 |
| Annotated in at least one Database | 55,581 | 55.46 |
| Total Unigenes | 102,036 | 100 |
