# Supplementary figures and images for: De novo assembly and annotation of the Amblyomma hebraeum tick midgut transcriptome response to Ehrlichia ruminantium infection
Source: PLoS Negl Trop Dis. 2023 Aug 14;17(8):e0011554. doi: 10.1371/journal.pntd.0011554 (PMC10449191; doi:10.1371/journal.pntd.0011554)

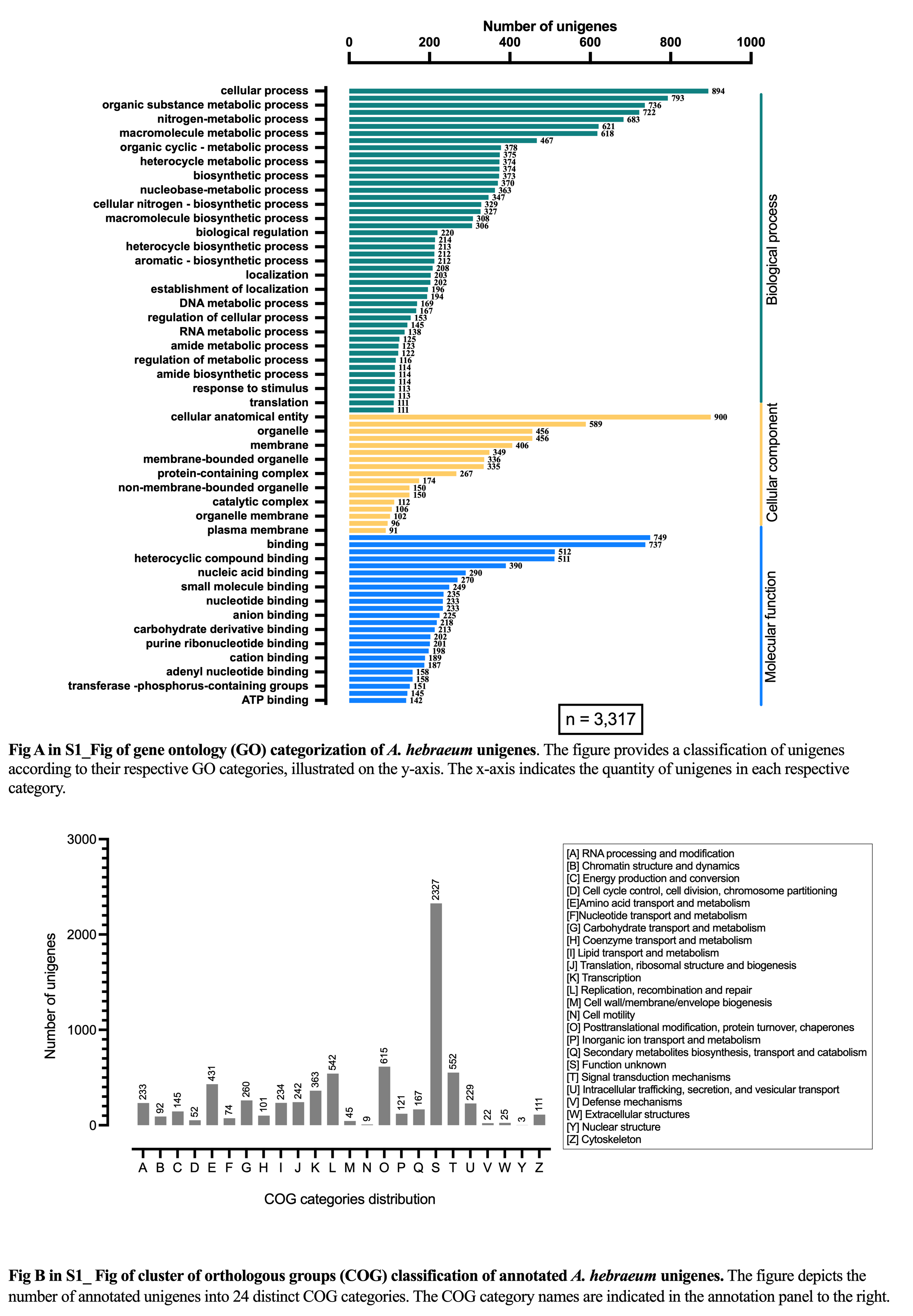

Supplement: S1 Fig — (TIFF) [file pntd.0011554.s003.tiff]
