# Supplementary material for: De novo assembly and annotation of the Amblyomma hebraeum tick midgut transcriptome response to Ehrlichia ruminantium infection
Source: PLoS Negl Trop Dis. 2023 Aug 14;17(8):e0011554. doi: 10.1371/journal.pntd.0011554 (PMC10449191; doi:10.1371/journal.pntd.0011554)
Supplement: S2 Data — (DOCX) [file pntd.0011554.s005.docx]

Supplementary Material

*De novo* assembly and annotation of the *Amblyomma hebraeum* tick midgut transcriptome response to *Ehrlichia ruminantium* infection

David Omondi^*^, Erich Zweygarth, Edwin Murungi, Frans Jongejan, Ard M. Nijhof

*** Correspondence:** David Omondi: [domolbio@gmail.com](mailto:domolbio@gmail.com)

Table A in S2 data on comparison of DEGs values of 8 genes of *A. hebraeum* nymph midgut. The log2 ratios qPCR plotted against those of respective RNA-Seq.

| **Unigene ID** | **Blast_nr Annotation** | **FC** | **Log2FC qPCR** | **FC** | **Log2FC RNA-Seq** |
| --- | --- | --- | --- | --- | --- |
| trinity_SMG_CL5318Contig2_1 | XP_037268604.1 acanthoscurrin-2-like [*R. microplus*] | 680.28 | 9.41 | 2486.67 | 11.28 |
| trinity_SMG_CL77607Contig1_1 | XP_054934363.1 uncharacterized protein LOC126543 [*D. andersoni*] | 9.18 | 3.2 | 556.41 | 9.12 |
| trinity_SMG_CL233Contig5_1 | XP_037564726.1 acanthoscurrin-2-like [*D. silvarum*] | 136.23 | 7.09 | 404.50 | 8.66 |
| trinity_SMG_TRINITY_DN148906_c0_g1_i1_1 | XP_037558824.1 shematrin-like protein 2 [*D. silvarum*] | 29.85 | 4.9 | 333.14 | 8.38 |
| trinity_SMG_TRINITY_DN28987_c1_g2_i1_1 | XP_049268204.1 keratin-associated protein 21-1-like [*R. sanguineus*] | 46.21 | 5.53 | 172.44 | 7.43 |
| trinity_FM3_TRINITY_DN126090_c0_g1_i2_1 | XP_050031932.1 venom serine carboxypeptidase-like [*D. andersoni*] | 10.62 | 3.41 | 151.16 | 7.24 |
| trinity_MM0_TRINITY_DN34998_c0_g1_i4_1 | Unknown | 0.054 | -4.21 | 0.0016 | -9.22 |
| trinity_FM3_CL75372Contig1_1 | Unknown | 0.077 | -3.69 | 0.0012 | -9.68 |

| Primer Table B in S2 data on comparison of DEGs values of 8 genes of *A. hebraeum* unfed female midgut. The log2 ratios qPCR plotted against those of respective RNA-Seq | | | | | |
| --- | --- | --- | --- | --- | --- |
| **Unigene ID** | **Blast_nr Annotation** | **FC** | **Log2FC qPCR** | **FC** | **Log2FC RNA-Seq** |
| trinity_MM0_TRINITY_DN2641_c0_g1_i1_1 | unknown | 238.85 | 7.9 | 359.53 | 8.49 |
| trinity_SMG_CL34206Contig1_1 | ACF35524.1 putative legumain-like protease precursor [*D. variabilis*] | 184.82 | 7.53 | 116.16 | 6.86 |
| trinity_SMG_TRINITY_DN20196_c0_g2_i2_1 | XP_049527598.1 LOW QUALITY PROTEIN: histone H3 [*D. silvarum*] | 7.36 | 2.88 | 71.51 | 6.16 |
| trinity_SMG_TRINITY_DN2863_c0_g1_i9_1 | ABI74752.1 amercin [*A. americanum*] | 3.81 | 1.93 | 25.28 | 4.66 |
| trinity_FM3_TRINITY_DN2009_c0_g1_i10_1 | XP_050027273.1 inositol oxygenase-like [*D. andersoni*] | 0.239 | -2.06 | 0.069 | -3.85 |
| trinity_FM0_SCL2340Contig1_1 | XP_037526299.1 TNF receptor-associated factor 6 [*R. sanguineus*] | 0.140 | -2.83 | 0.068 | -3.87 |
| trinity_MM2_CL956Contig2_1 | XP_049512374.1 tenascin-R isoform X2 [*D. silvarum*] | 0.134 | -2.89 | 0.068 | -3.87 |
| trinity_MM0_TRINITY_DN34998_c0_g1_i4_1 | unknown | 0.024 | -5.35 | 0.062 | -3.99 |

Table C in S2 data on comparison of DEGs values of 8 genes of *A. hebraeum* unfed male midgut. The log2 ratios qPCR plotted against those of respective RNA-Seq

| **Unigene ID** | **Blast_nr Annotation** | **FC** | **Log2FC qPCR** | **FC** | **Log2FC RNA-Seq** |
| --- | --- | --- | --- | --- | --- |
| trinity_MM2_TRINITY_DN50722_c0_g1_i9_1 | unknown | 65.79 | 6.04 | 458.25 | 8.84 |
| trinity_SMG_TRINITY_DN6410_c0_g1_i17_1 | unknown | 96.33 | 6.59 | 347.29 | 8.44 |
| trinity_SMG_TRINITY_DN20196_c0_g2_i2_1 | XP_049527598.1 histone H3 [*D. silvarum*] | 8.63 | 3.11 | 78.24 | 6.29 |
| trinity_FM0_TRINITY_DN13079_c0_g1_i1_1 | AAR97292.1 hebreain [*A. hebraeum*] | 4.99 | 2.32 | 43.41 | 5.44 |
| trinity_FM3_TRINITY_DN18829_c0_g1_i3_1 | SCV66484.1 Reverse transcriptase (RNA-dependent DNA polymerase) | 3.71 | 1.89 | 15.13 | 3.92 |
| trinity_FM3_SCL11Contig604_1 | KAG0428456.1 hypothetical protein HPB47_024567 [*I. persulcatus*] | 0.281 | -1.83 | 0.100 | -3.31 |
| trinity_MM0_TRINITY_DN131493_c0_g1_i1_1 | unknown | 0.129 | -2.95 | 0.037 | -4.74 |
| trinity_FM0_TRINITY_DN87676_c0_g1_i2_1 | unknown | 0.011 | -6.52 | 0.028 | -5.12 |

Table D in S2 data on comparison of DEGs values of 8 genes of *A. hebraeum* partly-fed female midgut. The log2 ratios qPCR plotted against those of respective RNA-Seq

| **Unigene ID** | **Blast_nr Annotation** | **FC** | **Log2FC qPCR** | **FC** | **Log2FC RNA-Seq** |
| --- | --- | --- | --- | --- | --- |
| trinity_FM3_TRINITY_DN71869_c0_g3_i1_1 | unknown | 48.84 | 5.61 | 284.04 | 8.15 |
| trinity_SMG_TRINITY_DN19612_c2_g4_i1_1 | KAH7939345.1 hypothetical protein HPB52_011352 [*R. sanguineus*] | 114.56 | 6.84 | 93.05 | 6.54 |
| trinity_SMG_TRINITY_DN2863_c0_g1_i9_1 | ABI74752.1 amercin [Amblyomma americanum] | 280.13 | 8.13 | 69.07 | 6.11 |
| trinity_FM3_TRINITY_DN3711_c0_g1_i8_1 | XP_050045023.1 steroid 17-alpha-hydroxylase/17,20 lyase-like [*D. andersoni*] | 149.08 | 7.22 | 31.77 | 4.99 |
| trinity_SMG_TRINITY_DN839_c0_g1_i14_1 | XP_037562278.1 uncharacterized protein LOC119441739 [*D. silvarum*] | 14.32 | 3.84 | 19.42 | 4.28 |
| trinity_FM3_TRINITY_DN4923_c3_g1_i1_1 | ABI74752.1 amercin [*A. americanum*] | 3.89 | 1.96 | 16.56 | 4.05 |
| trinity_FM3_TRINITY_DN116440_c0_g1_i2_1 | DAA34636.1 TPA_inf: hypothetical secreted protein 1496 [*A. variegatum*] | 0.192 | -2.38 | 0.078 | -3.68 |
| trinity_MM2_TRINITY_DN307197_c0_g1_i1_1 | XP_050036064.1 neuropeptide-like protein 31 [*D. andersoni*] | 0.046 | -4.44 | 0.070 | -3.83 |
| trinity_FM0_TRINITY_DN21546_c0_g1_i1_1 | DAA34694.1 TPA_inf: peritrophin [*A. variegatum*] | 0.196 | -2.35 | 0.057 | -4.11 |

Table E in S2 data on comparison of DEGs values of 8 genes of *A. hebraeum* partly-fed male midgut. The log2 ratios qPCR plotted against those of respective RNA-Seq

| **Unigene ID** | **Blast_nr Annotation** | **FC** | **Log2FC qPCR** | **FC** | **Log2FC RNA-Seq** |
| --- | --- | --- | --- | --- | --- |
| trinity_MM0_CL40198Contig1_1 | unknown | 15.34 | 3.94 | 153.27 | 7.26 |
| trinity_SMG_CL4330Contig1_1 | XP_054932033.1 uncharacterized protein LOC126540486 [*D. andersoni]* | 40.50 | 5.34 | 75.06 | 6.23 |
| trinity_MM2_TRINITY_DN17428_c0_g1_i2_1 | XP_037284856.1 uncharacterized protein LOC119177773 iso X4 [*R. microplus*] | 362.03 | 8.5 | 30.06 | 4.91 |
| trinity_FM3_TRINITY_DN22490_c0_g1_i1_1 | XP_050043259.2 uncharacterized protein LOC126540484 [*D. andersoni*] | 3.18 | 1.67 | 28.05 | 4.81 |
| trinity_MM2_TRINITY_DN56378_c0_g1_i1_1 | XP_050047744.2 uncharacterized protein LOC126544455 [*D. andersoni*] | 4.85 | 2.28 | 23.42 | 4.55 |
| trinity_MM2_TRINITY_DN3_c12_g1_i1_1 | unknown | 0.039 | -4.66 | 0.0083 | -6.9 |
| trinity_SMG_TRINITY_DN12092_c3_g1_i1_1 | unknown | 0.078 | -3.67 | 0.0050 | -7.63 |
| trinity_SMG_CL8950Contig1_1 | unknown | 0.0021 | -8.86 | 0.0042 | -7.88 |
